# Foundations for pediatric vocal biomarkers: age-aware phoneme recognition and latent-space error analysis

**DOI:** 10.3389/fnhum.2026.1802703

**Published:** 2026-08-04

**Authors:** Chethana Saligram, Vishal Shrivastava, Marisha Speights

**Affiliations:** Roxelyn and Richard Pepper Department of Communication Disorders, Northwestern University, Evanston, IL, United States

**Keywords:** child speech modeling, coarticulation dynamics, latent acoustic trajectories, pediatric speech, phoneme error rate, phoneme recognition, speech sound disorder (SSD), vocal biomarkers

## Abstract

**Introduction:**

Pediatric speech sound disorders (SSDs) affect many young children and are commonly assessed through auditory-perceptual judgments and IPA transcription, which are limited by listener bias, variable interrater reliability, and difficulty attributing deviations at the phoneme level. We present a developmentally informed framework for pediatric vocal biomarker foundations that prioritizes age-aware, phoneme-resolved interpretability over utterance-level accuracy alone.

**Methods:**

Using a CAAP-derived subset of the SEED corpus (27–94 months) with clinically specified phoneme targets and SSD labels, we construct age-stratified phoneme profiles and quantify error structure via phoneme error rate (PER) and age-banded confusion signatures. We operationalize co-articulation as transition dynamics from MFCC trajectories and their first and second derivatives, reflecting the velocity and acceleration of spectral change across adjacent segments. We learn compact acoustic representations with a variational autoencoder (VAE) and model temporal evolution with BiLSTMs, including attention, to characterize disorder-relevant instability in latent trajectories. For phoneme transcription, we train BiLSTM-CTC sequence models on clinically elicited speech and evaluate disorder classification and phoneme substitution patterns within age groups, then stress-test generalization on ECSC "Frog Story" narratives from TalkBank/CHILDES.

**Results:**

Co-articulation transition magnitudes varied systematically by age and clinical group. Attention improved temporal classification specifically in younger children, and PER dropped markedly between ages 3–4 and 4–5. On long-form naturalistic narratives, CTC training and inference remained numerically stable, but greedy decoding produced degenerate output dominated by blank and repetition tokens.

**Discussion:**

Together, these results support an age-aware approach to pediatric phoneme analytics, in which articulatory coordination, not just phoneme identity, carries developmentally and clinically relevant information; and phoneme-level performance should be interpreted relative to developmental stage rather than a single fixed benchmark. The decoding failures on naturalistic speech indicate a bottleneck specific to decoding rather than to the underlying representations, motivating constrained, hierarchical, or duration-aware decoding as a tractable next step. These findings position age-stratified, phoneme-resolved analysis as a foundation for interpretable and scalable pediatric screening and biomarker development.

## 1 Introduction

Pediatric speech sound disorders (SSDs) are estimated to affect approximately 8%–9% of young children (NIDCD, 2025). Speech acquisition requires children to master complex, coordinated movements of the articulators (e.g., jaw, lips, and tongue) within the vocal tract and to refine articulatory gestures during early development (MacNeilage et al., 2000; Namasivayam et al., 2025; Vorperian et al., 2005). SSDs are characterized by persistent difficulties in producing the speech sounds of a language and reflect disruptions in the integration of phonological representations (phonemes as abstract contrastive categories) with the speech-motor planning and coordination required to achieve acoustic-articulatory targets under a language's phonotactic constraints (Namasivayam et al., 2025; Association, 2025). In this paper, we evaluated candidate pediatric articulatory and linguistic vocal biomarkers as quantitatively derived speech measures that make phoneme-level production structure observable and comparable across children (Pizzimenti et al., 2025).

Multidimensional speech factors, including the sequencing of neuromuscular activations under spatial and temporal constraints, are shaped during early development. This dynamic interaction yields phoneme productions that are realized through context-dependent coarticulation (Dodd, 2014; Hua and Dodd, 2000; Ma et al., 2022; Namasivayam et al., 2020). Mastery of oral articulatory skills progress along a nonlinear developmental trajectory and at different rates in children (Kent, 1996; Locke, 1983; Kent, 1992; Namasivayam et al., 2020; MacNeilage et al., 2000). When a child's articulatory movements and speech sounds resemble those seen at earlier stages of development, it may reflect a complex underlying difficulty with speech sound production. Collectively, these developmental dynamics motivate multidimensional approaches for characterizing children's speech production patterns with sufficient granularity. However, in practice, pediatric speech assessment and research pipelines still rely heavily on listener-based auditory judgments and the use of the International Phonetic Alphabet (IPA) (Association, 1999) transcription as the primary measurement substrate. This dominant measurement approach is methodologically insufficient because coarticulation and rapid maturational change yield continuous, context-dependent realizations that must be collapsed into discrete transcription symbols and boundary decisions (Namasivayam et al., 2025).

Critically, sole reliance on perceptual IPA transcription raises a central ground-truthing challenge: it requires inferences about speech production to be drawn from surface-level phonetic evidence (what is heard and transcribed), even though the clinically relevant constructs implicate latent processes (phonological representations, planning/programming, and motor execution) that are not uniquely identifiable from surface forms (Kent, 1996; Kearney and Guenther, 2019; Kröger et al., 2022). In other words, IPA transcriptions encode realized phones and their perceived deviations, but they are still an indirect measurement that is subject to perceptual bias, listener normalization, and has limited reliability for subtle contrasts and short-duration events (Namasivayam et al., 2025). Consequently, human transcriptions alone cannot capture the full multidimensionality of speech production, and exclusive reliance on them as evidentiary ground truth is brittle rather than robust (Munson et al., 2010; Shriberg et al., 2010).

These limitations motivate automated approaches; however, child speech differs systematically from adult speech along developmental, acoustic, and articulatory dimensions that challenge conventional automatic speech recognition (ASR) systems (Bhardwaj et al., 2022; Gerosa et al., 2009; Shahin et al., 2019). Age-dependent variability in vowel quality, coarticulation, prosody (e.g., rate, duration, and pitch), and immature motor control can degrade recognition performance and obscure clinically meaningful deviations when models are trained primarily on adult speech (Potamianos and Narayanan, 2004; Yeung and Alwan, 2018). Errors arise not only from acoustic variability but also from linguistic assumptions embedded in adult-trained models, which can normalize or over-correct non-adult productions. As a result, systems optimized for adult transcription may suppress precisely the deviations clinicians aim to detect. Moreover, many automated tools emphasize word- or utterance-level transcription accuracy, which is misaligned with clinical questions that require phoneme-level attribution: which specific phonemes deviate, how frequently, in what direction, and whether these deviations exceed age-expected variability.

A second barrier to translation is interpretability. For pediatric applications, interpretability is not ancillary: clinically useful outputs must be segment-resolved, developmentally contextualized, and verifiable. Black-box recognition models that provide only aggregate accuracy offer limited insight into why errors occur, whether they reflect developmentally plausible variability, or whether they indicate disorder-relevant deviations (Zhang et al., 2021; Rudin, 2019; Benway and Preston, 2025). A clinically meaningful phoneme-level pipeline should therefore (1) explicitly represent developmental variability, (2) quantify phoneme-resolved error structure rather than only overall accuracy, and (3) incorporate uncertainty- or deviation-sensitive signals that distinguish ambiguous productions from systematic misarticulation patterns (Xu et al., 2025; Berisha and Liss, 2024).

Recent advances in self-supervised learning (SSL) provide a technical basis for improving sensitivity to pronunciation variation in pediatric speech. SSL models such as wav2vec 2.0 learn contextualized acoustic representations from raw audio without manual labels (Baevski et al., 2020) and have been shown to capture fine-grained pronunciation differences that correlate more strongly with human similarity judgments than conventional handcrafted features such as MFCCs or transcription-derived edit distances (Mahendran et al., 2023; Tirronen et al., 2022). Intermediate SSL layers, in particular, often encode generalizable acoustic-phonetic structure (Binder et al., 2016; Pasad et al., 2023), making them promising substrates for segment-sensitive analyses. However, a key gap remains: these representations and ASR outputs are rarely translated into developmentally grounded, phoneme-level error analytics that are interpretable and actionable for pediatric screening and SSD risk characterization. Bridging this gap requires analytic frameworks that map learned representations to interpretable, phoneme-level evidence by pairing explicit phoneme sequence recognition with complementary latent deviation modeling, so that deviation can be quantified in a way that is both segment-resolved and uncertainty-sensitive. In the present work, this analytic mapping is instantiated using MFCC-based features as a transparent and well-characterized acoustic measurement substrate, rather than as a claim of representational optimality. This choice enables explicit attribution of phoneme-level error structure and coarticulation dynamics across age, while remaining agnostic to the specific front-end representation and compatible with future extension to self-supervised embeddings.

With this analytic perspective in place, we develop and evaluate a developmentally informed, phoneme-level recognition and error analysis framework for pediatric speech. Using age-stratified child speech with phoneme-level targets, we implement a recurrent sequence model for phoneme recognition from single-word and continuous speech. The model is trained with a monotonic-alignment objective that avoids the need for manually segmented phoneme boundaries, using a BiLSTM acoustic model with connectionist temporal classification (CTC) to produce phoneme sequence predictions. CTC provides a principled objective for learning from unsegmented sequences by marginalizing alignments, and it remains a standard choice for end-to-end speech sequence learning when explicit boundaries are unavailable (Graves et al., 2006). In parallel, we learn complementary latent representations using a variational autoencoder (VAE) to support analyses that are less dependent on categorical transcript agreement (Kingma and Welling, 2013; Girin et al., 2022). Guided by three research questions, we evaluate: (1) how accurately the model predicts phoneme sequences in clinically elicited speech as quantified by phoneme error rate (PER) (Lopes and Perdigao, 2011) and age-stratified confusion structure; (2) whether coarticulation dynamics, operationalized via transition-based statistics, provide additional discriminatory and interpretable information beyond segmental outcomes; and (3) the extent to which models developed on structured clinical speech remain robust when applied to long-form, naturalistic child narratives without phoneme-level ground truth. Together, these analyses aim to preserve clinically relevant detail while producing interpretable, developmentally contextualized summaries that enable scalable phoneme-level measurement.

## 2 Methodology

### 2.1 Ethics

This study is a secondary analysis of previously collected pediatric speech recordings and associated annotations. Clinical recordings were collected and made available by authors under IRB approval with parent/guardian informed consent (and child assent when applicable), including permission for secondary use of child speech data without personal identifiers for child speech research studies. We use two complementary pediatric speech datasets that differ in elicitation and annotation structure: (1) the Speech Exemplar and Evaluation Database (Speights Atkins et al., 2020), a corpus of clinically elicited speech with phoneme-level targets and error coding to support developmentally contextualized phoneme error profiling and (2) the Eugene Children's Story Corpus (ECSC) “Frog Story” dataset from TalkBank/CHILDES (Kallay and Redford, 2020), used to evaluate robustness of phoneme sequence modeling. Methods for data collection and recordings are detailed in the respective manuscripts.

### 2.2 Data

#### 2.2.1 Clinical CAAP-derived dataset (SEED corpus subset)

The primary clinical dataset used in this study is derived from CAAP (Clinical Assessment of Articulation and Phonology) (Secord and Donohue, 2002) recordings within the SEED developmental speech corpus. The dataset includes children aged 27 to 94 months (2.3 to 7.8 years). Speech health status was defined in the parent SEED study using CAAP standard scores, with scores ≤ 80 (more than one standard deviation below the mean of 100) classified as SSD-present (SSD-P) and ≥85 classified as SSD-not present (SSD-NP). Recordings consist primarily of short, clinically elicited productions with constrained lexical content. The CAAP dataset comprised a total of 789 speech samples distributed across six age bands: 29 from children aged 2–3 years; 133 from 3–4 years; 417 from 4–5 years; 90 from 5–6 years; 75 from 6–7 years; and 45 from 7–8 years. Although the CAAP subset spans six age bands, the present modeling analyses focus specifically on the 3–4 year and 4–5 year bands. We introduced age-band as a group descriptor to acknowledge that development occurs incrementally and non-linearly across the months within an age-band; however, due to small cell size, sub-grouping according to months was not feasible for this study. These two bands were selected because they contained sufficient representation of both SSD-P and SSD-NP samples to support balanced modeling and group-level comparison. In the remaining age bands, class distribution was either sparse or insufficiently balanced for reliable statistical and model-based analysis. Accordingly, all transition modeling, confusion matrix analyses, and diagnostic evaluations reported in this paper are restricted to the 3–4 and 4–5 year cohorts.

**Figure 1:**
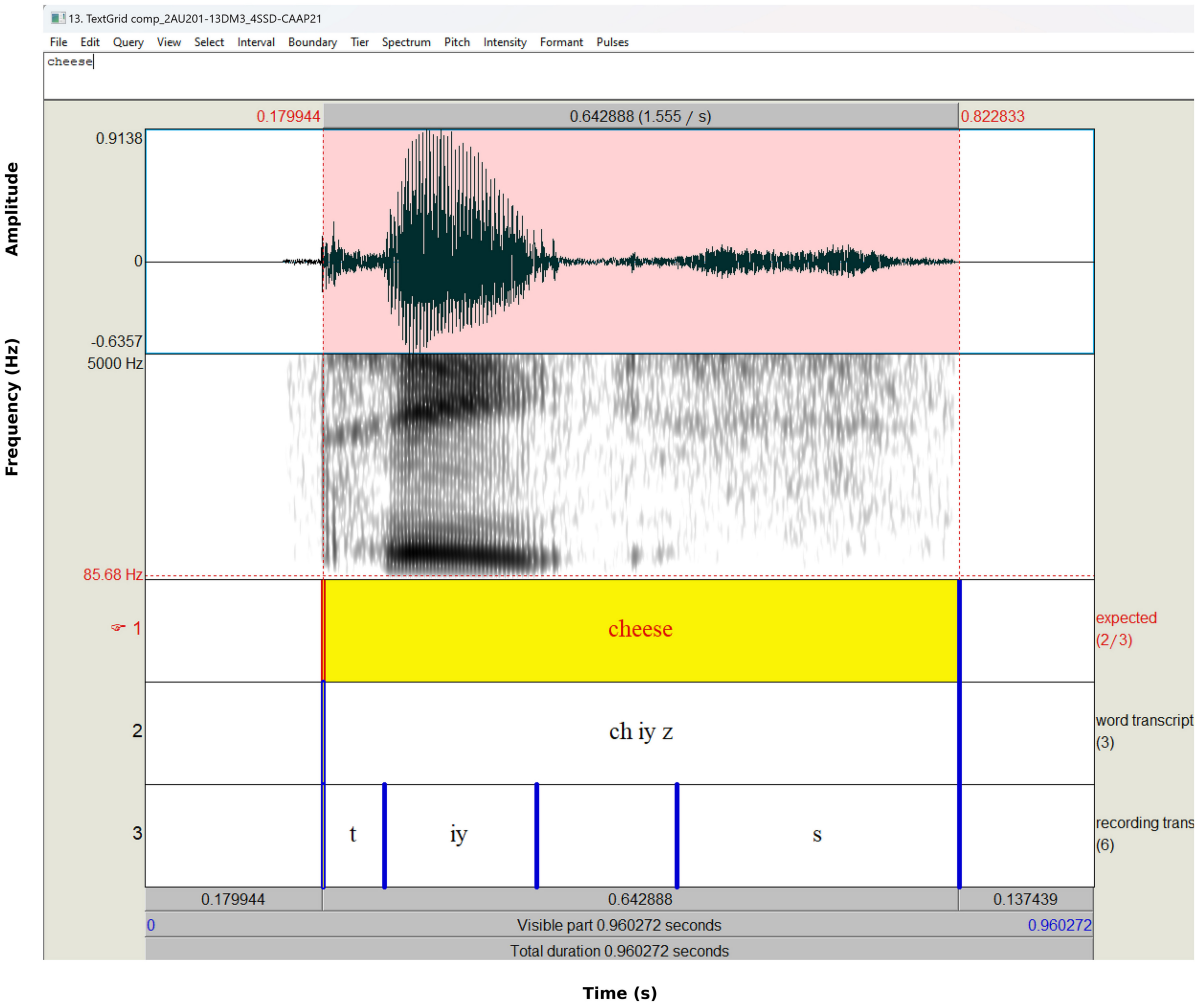
Example of produced speech from a child with SSD-present producing the word cheese with a word initial and word final sound errors.

Each recording includes phoneme-level targets and response coding that provide reference (ground-truth) phoneme sequences and categorical error labels (e.g., substitution/insertion/deletion). Analyses were stratified by age band to reduce confounding due to developmental variability. Annotations were completed by three trained research assistants who underwent academic and lab-based phonetic transcription training. Annotated phonemes and error code types (substitution, insertion, and deletion) were determined in a two-stage process: blind transcription, followed by a team transcription consensus procedure to adjudicate disagreements. The annotated dataset supports computation of phoneme error rate (PER), age-stratified confusion matrices, and downstream diagnostic modeling. An example waveform/spectrogram with aligned phoneme labels is shown in Figure 1.

#### 2.2.2 ECSC “Frog Story” narrative dataset (TalkBank/CHILDES)

To evaluate phoneme modeling under more naturalistic, multi-word conditions, we additionally used the Eugene Children's Story Corpus (ECSC) “Frog Story” dataset from TalkBank/CHILDES (Kallay and Redford, 2020). ECSC contains spontaneous, continuous narrative speech elicited using the Mercer Mayer's *Frog Where Are You* picture book (Berman and Slobin, 1994). The ECSC dataset includes 367 audio recordings with accompanying transcriptions, of which 341 are child narrative recordings from 188 children and 26 adult caregivers. In this study, only child recordings are used. The dataset does not include clinical diagnostic labels for SSD and speech sound disorder classification was not qualitatively determined from the ECSC recordings. The children were between 5 and 8 years old at first participation, with a subset followed longitudinally for up to three years (up to 7 to 10 years). Recordings are approximately 2 minutes in duration. Transcripts are sentence-level non-phonetically transcribed text (orthographic), without explicit frame-level or segment-level timing. ECSC does not provide reference phoneme sequences or phoneme boundary annotations; therefore, any phoneme sequences used in ECSC analyses are derived by the modeling framework (i.e., predicted phoneme sequences) rather than supplied as ground truth.

Together, these datasets provide complementary views of pediatric phoneme production. The CAAP-derived dataset supports clinically grounded phoneme error profiling and disorder-aware modeling. ECSC provides multi-word naturalistic speech and no annotated phoneme boundary annotations, requiring phoneme sequences to be derived from model outputs. In this study, ECSC was used to examine model behavior when applied to longer, variable-length recordings in the absence of phoneme-level reference sequences.

### 2.3 Setting the foundation for scalable measurement methods

This study is organized around three research questions that jointly define the measurement objectives, modeling scope, and evaluation strategy. Hypothesis testing and exploratory analyses are grounded in the following framework.

#### 2.3.1 RQ1: phoneme sequence prediction accuracy on clinically elicited speech (CAAP/SEED)

RQ1 asks whether a supervised phoneme recognition model can predict a child's phoneme sequence in clinically elicited speech (relative to reference phoneme annotations), and whether the model's error structure is consistent with age-stratified target-outcome patterns present in the clinical annotations. Analyses use the CAAP-derived subset of the SEED, which provides ground truth produced phoneme sequences and speech status labels.

Pediatric phoneme production varies non-uniformly with development; therefore, to establish a developmental baseline, all analyses were stratified by age band. For each age band and elicited target phonemes, we tabulated (1) the proportion of productions annotated as correct and (2) the empirical distribution of annotated outcomes, including substitution, insertion, and deletion events, and the most substituted phoneme outcomes. These summaries defined the age-referenced construct against which model-derived phoneme confusions are interpreted.

Within this framing, automated transcription was evaluated by comparing the model's predicted phoneme sequence against the annotated phoneme sequence. Phoneme accuracy was quantified using phoneme error rate (PER), defined as the normalized Levenshtein edit distance (Yujian and Bo, 2007) between predicted and reported by age band. To support interpretability, we additionally summarized reference-vs.-predicted confusion structure within each age-band and speech health subtype by tabulating reference to predicted phoneme mappings. It is hypothesized that SSD-NP speech will exhibit stronger diagonal dominance and constrained confusions, while SSD-P speech will show broader dispersion, consistent with the age-stratified annotation profiles. Deviations observed at older ages are further examined to assess whether they reflect atypical persistence or narrowing of confusions relative to developmental expectations. RQ1 was operationalized using Pipeline B (Section 3.5.2). Evaluation for RQ1 is based on PER and age-stratified confusion matrices computed on held-out CAAP samples, with planned reporting consisting of PER values presented alongside confusion visualizations directly comparable to the annotation-derived target–response matrices.

#### 2.3.2 RQ2: co-articulation/transition dynamics as a complementary measurement dimension

RQ2 asks whether co-articulation-derived transition measures capture systematic group- and age-related differences in CAAP speech, and whether these measures offer discriminative value that is not redundant with phoneme-sequence outcomes. RQ2 uses the same CAAP-derived subset and age-banded stratification as RQ1, but shifts focus from categorical sequence outcomes to continuous articulatory dynamics.

Co-articulation was operationalized as transition dynamics derived from MFCC trajectories and their first- and second-order temporal derivatives, which quantify the magnitude and stability of spectral change across adjacent phonetic regions. We first examine how transition magnitude distributions differ between SSD-P and SSD-NP children across age bands, establishing whether the latter speech shows stronger phoneme boundary contrast and whether this contrast increases with age. Specifically, we hypothesize that spectral transitions become more stable across development, consistent with improving articulatory control and clearer acoustic separation between adjacent phonetic segments. We test whether sequential modeling of transition trajectories supports disorder discrimination and how this varies with age. Finally, transition measures provide complementary evidence in cases where phoneme confusion patterns overlap, with differences in articulatory precision hypothesized despite similar categorical outcomes. Planned RQ2 analyses consist of age-stratified descriptive statistics (and, where applicable, classification performance metrics on held-out CAAP data). Reporting includes transition summary plots by age and speech status, followed by comparative results assessing incremental value relative to the RQ1 phoneme-transcription baseline.

#### 2.3.3 RQ3: robustness under long-form, naturalistic child narratives without phoneme-level ground truth (ECSC/Frog Story)

RQ3 asks whether models trained on short, clinically elicited CAAP utterances produce stable and interpretable outputs when applied to long-form narrative speech in the absence of phoneme-level sequences. RQ3 uses the ECSC “Frog Story” dataset, which lacks phoneme boundary annotations and reference phoneme sequences (i.e., has no ground-truth), and doesn't have any SSD health status. RQ3 is framed as an exploratory out-of-domain robustness evaluation rather than a supervised transcription benchmark.

To define the robustness challenge, we characterize differences in **temporal scale and utterance structure** between the CAAP-derived and ECSC datasets by summarizing recording duration and sentence-length distributions. The trained model is then applied to ECSC audio to generate predicted phoneme sequences and frame-level phoneme evidence over time. RQ3 evaluates whether inference remains numerically stable over long durations and examines qualitative characteristics of the outputs to identify mismatches between training and deployment conditions. Evaluation for RQ3 is based on decoding stability diagnostics, distributional properties of model outputs, and qualitative inspection of representative sequences. Planned reporting consists of representative narrative examples, summary diagnostics of decoding behavior, and documentation of observed failure modes without accuracy claims.

### 2.4 Acoustic measurement and feature extraction pipeline

**Figure 2:**
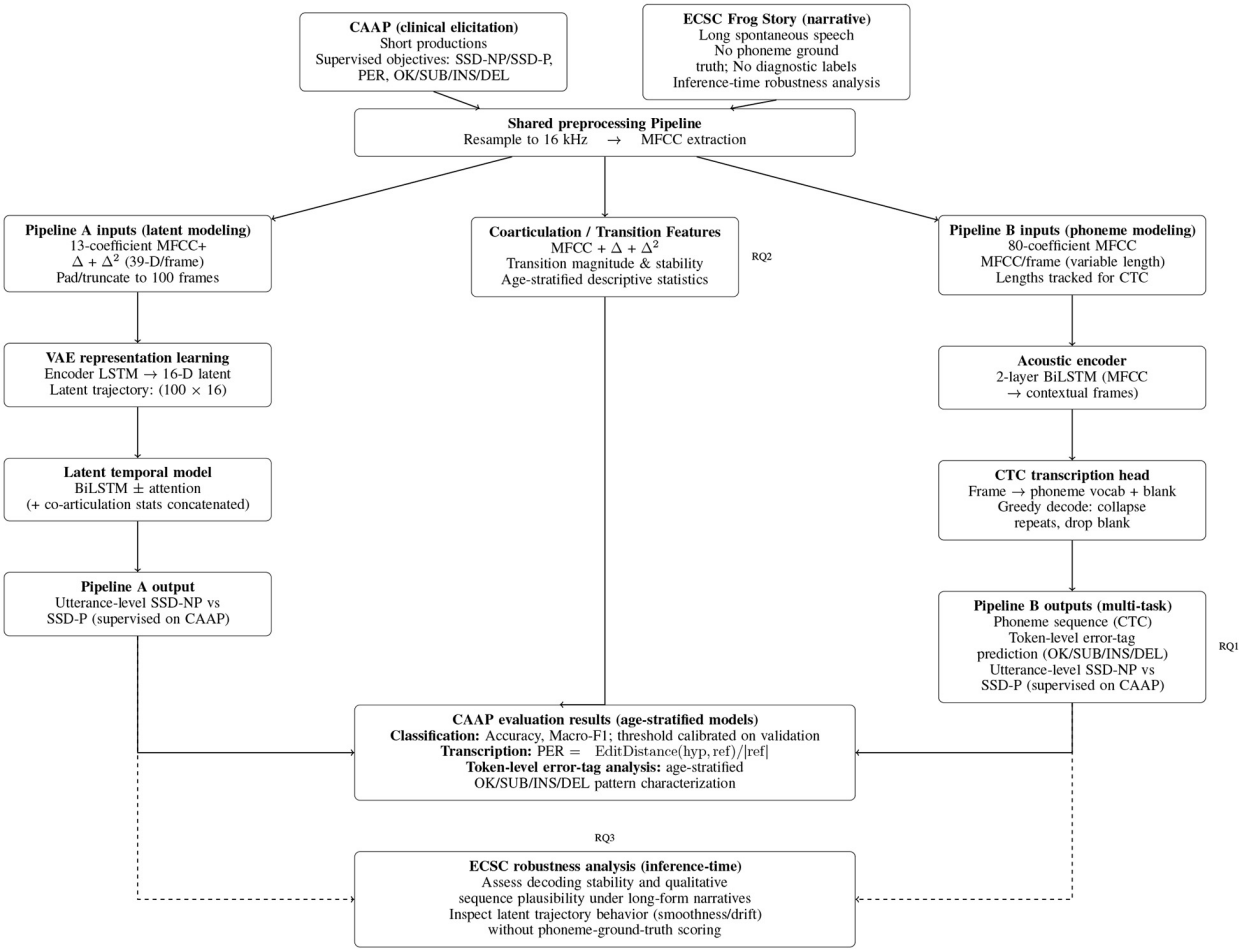
Overview of the measurement pipeline and modeling framework. Two pediatric speech datasets are used: clinically elicited CAAP recordings with phoneme-level targets and error annotations **(left)** and ECSC Frog Story narratives without phoneme-level ground truth **(right)**. A shared preprocessing stage extracts MFCC-based representations and feeds three analysis paths. Pipeline A performs latent acoustic modeling for utterance-level SSD classification and trajectory analysis. Pipeline B performs supervised phoneme sequence modeling with CTC for transcription, phoneme error characterization, and disorder classification. ECSC is used exclusively as an out-of-domain robustness probe to assess inference-time stability and the qualitative structure of decoded outputs.

The measurement pipeline is organized to mirror the three research questions and is summarized in Figure 2. We first define a shared preprocessing layer applied to all audio. We then describe (1) the phoneme sequencing pipeline used for RQ1, (2) the coarticulation pipeline used for RQ2, and (3) the out-of-domain robustness application and evaluation constraints for RQ3.

#### 2.4.1 Developmental baseline: age-stratified target-response profiling (CAAP)

To establish a developmental baseline for interpreting phoneme confusions independent of any learned model, we first computed age-stratified target-response profiles directly from the CAAP annotations. This step characterized the empirical error structure in children's speech productions relative to clinically specified elicitation targets prior to supervised learning, decoding, or inference. We introduced age-band as a group descriptor to acknowledge that development occurs incrementally and non-linearly across the months within an age-band.

Within each band, we aggregated all elicited instances for each target phoneme and tabulated the observed response outcomes recorded in the clinical alignment fields. For each target phoneme, we compute a row-normalized target → response distribution that summarizes (1) the proportion of productions coded as correct and (2) the empirical distribution of categorical outcomes, including substitutions, insertions, and deletion events. For substitution outcomes, we additionally summarized the most frequently substituted response phonemes within each age-band. Rows correspond to intended (target) phonemes and columns correspond to realized responses, yielding a target-response confusion matrix that represented the dataset's baseline phonological deviation structure.

#### 2.4.2 Shared pre-processing

All audio recordings were standardized through a unified pre-processing pipeline prior to any feature extraction and modeling. All waveforms were resampled to 16 kHz, converted to single-channel (mono) format, and amplitude-normalized to reduce signal variation. Leading and trailing non-speech segments were trimmed using an energy-based threshold while preserving within-utterance pauses that are characteristic of pediatric speech.

Because the two datasets differ substantially in temporal structure, duration normalization was performed in a task-aware manner. For representation learning and temporal modeling, CAAP utterances were converted into fixed-length sequences by zero-padding frame-level features to 100 time steps. This produces a consistent tensor shape while preserving the internal temporal structure of each word-level production. Padding masks were retained to prevent artificial contributions from non-speech frames during training. This fixed-window formulation was used exclusively for VAE and latent-space temporal modeling, and was not applied to ECSC narratives. For CTC-based phoneme transcription, variable-length sequences were preserved. Frame counts were tracked explicitly and passed to the loss function to ensure correct alignment between acoustic frames and symbolic targets. No temporal warping or forced segmentation was applied, allowing the model to learn directly from natural duration variability.

#### 2.4.3 Acoustic feature extraction

Each audio file was loaded, and its duration was computed as the total recording length in seconds. Overall amplitude characteristics were summarized using root-mean-square (RMS) energy, from which the mean and standard deviation values were computed to characterize average intensity and intensity variability. We also computed zero-crossing rate (ZCR), the rate at which the waveform changes sign across successive samples. ZCR provides a simple frame-level descriptor of signal noisiness and hig-frequency fluctuation, with higher values reflecting more rapid waveform oscillation. Mean and standard deviation were computed for each recording.

Spectral structure was summarized using Mel Frequency Cepstral Coefficients (MFCCs) and Mel-spectrogram statistics. MFCCs provide a compact representation of the spectral envelope using a perceptually motivated filterbank, and were computed by frame and summarized per-coefficient means and standard deviations. Because the two modeling pipelines operated at different temporal scales (fixed-length latent trajectory modeling vs. variable-length phoneme sequence modeling), MFCC framing parameters were pipeline specific. For Pipeline A (VAE-BiLSTM latent trajectory modeling) and the coarticulation transition features, MFCCs were extracted at 16 kHz using a 128 ms analysis window (*n*_*fft* = 2, 048) and a 32 ms hop length (hop_length = 512), yielding a 13-dimensional MFCC vector augmented with first- and second-order derivatives (Δ, Δ^2^; 39-dimensional total). For Pipeline B (CTC-based phoneme sequence modeling), MFCCs were extracted at 16 kHz using a 25 ms analysis window (*n*_*fft* = 400) with a 12.5 ms hop (hop_length = 200), yielding 80 coefficients per frame and retaining frame-length metadata for CTC.

#### 2.4.4 Voicing features and temporal measures

Fundamental frequency (F0) contours were estimated using Praat-based autocorrelation-based pitch tracking (via Parselmouth). From voice-frame F0 values, we computed mean F0 and standard deviation as indicators of intonational stability and modulation. For ECSC narratives, where speech is continuous and contains long unvoiced spans, abrupt register shifts, and variable speaking styles, F0 was extracted using the probabilistic YIN (pYIN) algorithm. pYIN formulates pitch estimation as a probabilistic inference problem, jointly estimating voiced/unvoiced states and fundamental frequency by modeling periodicity across frames. This allows it to remain stable in the presence of noise, breathy voice, and rapid prosodic variation that are common in spontaneous child speech.

#### 2.4.5 Rhythmic timing and energy peak structure

To approximate course rhythmic timing structure, we derived a short-time energy (STE) contour and applied a peak picking procedure to identify prominent syllable-like energy peaks. From the detected peaks, we computed (1) peak count, (2) mean inter-peak interval, and (3) inter-peak interval variability (standard deviation of inter-peak intervals). These measures were used as task-level descriptors of temporal patterning for short CAAP productions and were not intended to represent articulatory syllable boundaries. To characterize temporal regularity and voicing patterns, we computed voicing proportion and derived summary rhythm metrics (pitch-based rhythm, and energy-based rhythm) from framed audio. These features provide complementary, task-level descriptors of periodicity and temporal organization beyond segmental measures.

#### 2.4.6 Coarticulation and transition feature construction

To move beyond segment-level features and quantify how children connect, merge, or separate adjacent phonemes, we analyzed *co-articulation*—the continuous blending of phonemes that reflects articulatory planning and control for CAAP samples. This is developmentally sensitive, as children mature, transitions between phonemes become sharper, more stable, and more temporally consistent. Abnormalities in these transitions include blurred boundaries, reduced acoustic contrast, and unstable temporal progression, all of which are markers of decreased motor speech ability. This motivated the extraction of transition-focused acoustic measures derived from MFCC sequences and their temporal derivatives.

For each CAAP utterance, we computed MFCCs and their first- and second-order temporal derivatives (Δ and Δ^2^) on framed audio. MFCCs provide a compact representation of the spectral envelope, while Δ and Δ^2^ capture the instantaneous velocity and acceleration of spectral change. We operationalized “transition magnitude” as the frame-to-frame change in these trajectories, producing a time series that reflects how rapidly the spectrum evolves over the utterance.

From this transition-magnitude series, we computed task-level summary descriptors (e.g., central tendency and dispersion) to characterize overall co-articulatory sharpness and stability within each production. Transition features were analyzed within age bands to preserve developmental comparability. For each age band, we summarized transition magnitudes separately for the SSD-NP and SSD-P groups and examined distributional properties (e.g., location, spread, and overlap).

Because the transition signal is inherently sequential, we treated transition magnitude both as (1) a distribution summarized by descriptive statistics and (2) a time series that preserved within-utterance dynamics. This dual representation supports analyses that distinguish global differences in transition strength from differences in temporal patterning (e.g., smooth vs. irregular trajectories).

### 2.5 Modeling framework

Consistent with our framing, we evaluated candidate vocal biomarkers at three levels: phoneme-sequence error structure (PER and confusion profiles), transition-based coarticulation dynamics derived from MFCC trajectories, and temporal organization of learned acoustic representations. Analyses combined descriptive group comparisons with complementary sequence modeling stages designed to capture both developmental acoustic structure and clinically meaningful phonological outcomes in pediatric speech. Group-level statistical comparisons are conducted using two-sided independent-samples t-tests and are interpreted descriptively, reflecting their exploratory role and their use alongside model-based analyses rather than as standalone inferential claims.

We implement two complementary sequence modeling pipelines on CAAP. Pipeline A learns low-dimensional latent acoustic trajectories and models their within-utterance dynamics to study temporal structure in pediatric speech and to provide complementary utterance-level disorder sensitivity (SSD-P vs. SSD-NP) independent of explicit phoneme decoding. Importantly, Pipeline A is not designed to answer a standalone research question. Instead, it functions as a supporting analysis that contextualizes the phoneme- and transition-based results by testing whether disorder-relevant structure is present in global acoustic–temporal organization independent of explicit phoneme identity. This framing prevents over-attribution of phoneme-level findings to transcription artifacts and provides a representation-level control for subsequent analyses.

Pipeline B performs supervised phoneme sequence modeling to estimate produced phoneme strings from elicited speech and to support downstream confusion and error-event analyses relative to age-stratified clinical baselines, thereby directly addressing RQ1. Although both pipelines operate over the same underlying acoustic material, they differ in supervision and in the level at which structure is modeled (latent trajectory vs. explicit phoneme sequence). Both modeling pipelines are subsequently examined under out-of-domain robustness constraints on ECSC Frog Story narratives at inference time (RQ3); evaluation procedures and robustness analyses are described in Section 3.6.

Coarticulation and transition measures derived in Section 3.4.6 constitute a separate, explicit analysis stream addressing RQ2 and are incorporated into modeling in two ways: as optional summary descriptors concatenated to latent trajectories to inject interpretable coarticulatory information, and as transition-magnitude time series modeled directly by recurrent architectures to assess whether articulatory dynamics provide a discriminatory signal beyond static summaries.

#### 2.5.1 Pipeline a: latent acoustic trajectory and disorder modeling (VAE–BiLSTM)

Building on the acoustic and co-articulatory measurements defined in Section 3.4, we introduce *Pipeline A*, a two-stage modeling approach designed to analyze developmental and disorder-related temporal structure in pediatric speech. Pipeline A is used as a supporting analysis to probe whether disorder-relevant information is expressed in the global temporal organization of pediatric speech independent of explicit phoneme transcription. Rather than targeting phoneme-level accuracy or co-articulation metrics directly, this pipeline tests whether latent acoustic trajectories alone support utterance-level discrimination between SSD-not-present (SSD-NP) and SSD-present (SSD-P) speech. In this role, Pipeline A complements—but does not replace—the phoneme- and transition-focused analyses by establishing whether an SSD signal exists upstream of categorical phoneme outcomes.

Given the high acoustic variability of children's productions, Pipeline A first learns compact latent representations that suppress nuisance variability and then models how these representations evolve to support utterance-level disorder classification. Specifically, a Variational Autoencoder (VAE) is used to derive low-dimensional latent embeddings from MFCC-based inputs, which are subsequently processed by a Bidirectional LSTM (BiLSTM) to capture within-utterance temporal dynamics. Both components are trained on CAAP data; ECSC narratives are used only as an out-of-domain probe to assess inference-time stability and representation behavior under long-form, naturalistic speech, without supervised disorder evaluation. This pipeline focuses on latent temporal dynamics and is complementary to the phoneme-level sequence modeling pipeline introduced later, which addresses explicit phoneme transcription and error characterization.

Our earlier analyses motivate three design objectives that directly inform the architecture of Pipeline A:

1. **Reduce acoustic nuisance variability**. Pediatric speech exhibits high within- and between-speaker variability unrelated to disorder status. This is addressed by a variational autoencoder that enforces a low-dimensional, regularized latent space, encouraging invariant representations of acoustic structure.
2. **Model disorder cues expressed over time**. Many clinically meaningful differences manifest as instability or degradation across phonetic transitions rather than as static frame-level deviations. This motivates temporal modeling of latent trajectories using a Bidirectional LSTM, which captures within-utterance context and long-range dependencies.
3. **Maintain robustness beyond elicited speech**. Models trained on short, controlled CAAP utterances must operate plausibly on longer, unconstrained narratives. Pipeline A therefore decouples representation learning from lexical supervision and is evaluated on ECSC narratives as an out-of-domain probe of inference-time stability under long-form speech.

##### 2.5.1.1 Variational autoencoder for latent acoustic representation

The first stage uses a Variational Autoencoder (VAE) to map high-dimensional acoustic features to a compact, regularized latent space intended to be robust to the high variability characteristic of pediatric speech. Unlike deterministic autoencoders that learn a fixed latent code, VAEs learn a continuous probabilistic latent representation parameterized by mean and variance and trained using a reparameterization trick, which encourages smoothness and supports generalizable latent structure across heterogeneous inputs [35] [36]. Prior work has shown that VAE-style objectives can disentangle factors of variation in sequential speech data (e.g., speaker and content-related structure) and yield latent embeddings that are useful for downstream modeling (Hsu et al., 2017). VAE's have also been used for speech representation learning in setttings where robust latent features support classification and related tasks under variability (Latif et al., 2018), consistent with our use of a latent trajectory prior to disorder modeling.

Each frame is represented by 13 MFCC coefficients augmented with first- and second-order temporal derivatives (Δ, Δ^2^), yielding a 39-dimensional vector per frame.

##### 2.5.1.2 BiLSTM modeling of temporal dynamics in latent space

After learning latent acoustic representations with the VAE, we modeled how these representations evolve over time to perform utterance-level disorder classification. Clinically meaningful differences in motor speech precision often manifest in the temporal organization of latent trajectories, particularly around transitions where articulatory control is unstable.

A bidirectional LSTM (BiLSTM) is used to capture within-utterance temporal context by processing latent sequences in both forward and backward directions. This allows the model to integrate information from earlier and later portions of an utterance, which is important when disorder-related cues are distributed unevenly over time rather than localized to a single region. Bidirectionality further accommodates variable articulation rates and diffuse boundaries that characterize pediatric speech, enabling the classifier to model transition-related dynamics without assuming fixed temporal alignment.

For CAAP, the 16-dimensional latent vectors produced by the VAE are organized into fixed-length sequences of 100 time steps, yielding inputs of shape (*N*, 100, 16) for the BiLSTM. To enrich the temporal representation with explicit articulatory information, each latent sequence is optionally augmented with summary coarticulation descriptors derived in Section 3.4.6 (including the mean, standard deviation, extrema, and range of transition magnitude). These descriptors are concatenated with the latent representations prior to classification, allowing the model to jointly leverage spectral-dynamic structure from the latent space and articulatory-dynamic cues from transition measures. The BiLSTM encodes each augmented latent trajectory into a sequence-level representation that is used to predict SSD-NP vs. SSD-P speech at the utterance level.

To allow the model to emphasize diagnostically salient regions within each trajectory, we additionally evaluate an attention-augmented BiLSTM variant. The attention mechanism computes a normalized weight over hidden states and forms a weighted temporal summary, replacing uniform temporal pooling as input to the classification head. This allows brief but informative segments, such as transient transition instabilities or prolonged low-contrast regions, to contribute disproportionately to the final utterance-level representation and enables direct assessment of whether disorder-related cues in pediatric speech are temporally sparse and benefit from selective focus.

##### 2.5.1.3 Training setup for VAE-BiLSTM pipeline

Data are stratified by age band and partitioned into disjoint training (80%), validation (10%), and test (10%) splits at the recording level to ensure developmental homogeneity and prevent leakage across evaluation stages. Each age band is trained independently to avoid confounding developmental variability with disorder-related structure. Training uses:

- Adam optimizer with an initial learning rate of 3 × 10^−4^ and batch sizes of 32 (training) and 64 (validation/testing),
- categorical cross-entropy loss for utterance-level SSD classification, with inverse-frequency class weighting applied when age-band imbalance is present,
- ReduceLROnPlateau learning-rate scheduling to stabilize convergence under high-variance pediatric data.

Models are trained for 25 epochs on CAAP. The pipeline is intentionally structured to support evaluation on ECSC Frog Story narratives as a robustness stress test under naturalistic conditions:

- VAE embeddings provide a compact acoustic representation that is not tied to CAAP's fixed lexical structure,
- BiLSTM temporal modeling operates on variable-length latent trajectories, enabling application to long-form speech,
- Co-articulation statistics remain interpretable across both structured elicitation and spontaneous narrative speech.

This unified pipeline, therefore, serves both as a controlled modeling framework for CAAP and as an explicit probe of how pediatric speech models trained on clinical data behave when exposed to long, unaligned real-world child speech in ECSC.

#### 2.5.2 Pipeline B: phoneme sequence modeling with BiLSTM–CTC

Building on the established acoustic and co-articulatory structure of the CAAP dataset, we next train a supervised phoneme transcription model to predict children's realized phoneme sequences from acoustic features. The goal is not only to transcribe phonemes accurately but to do so in a way that is sensitive to developmental norms, co-articulation timing, and the characteristic error patterns of speech sound disorders. Pipeline B produced two concrete outputs used in Pipeline A: (1) a decoded phoneme sequence for each utterance (used to compute PER and error structure) and (2) auxillary predictions (error-event tags and utterance-level SSD probability) used for interpretive analysis. Section 3.6.1 specifies the decoding procedures that converted model logits into evaluated outputs.

##### 2.5.2.1 Preparing supervised phoneme targets

The phoneme labels for each audio sample were created by taking the child's spoken phoneme string, splitting it into individual phoneme symbols, and converting each symbol into a numeric identifier using a phoneme-to-index dictionary. This process produced a variable-length sequence of integers for every recording, which served as the supervised target sequence for training.

For each recording, the child-produced phoneme string was tokenized and mapped to integer identifiers using a phoneme-to-index dictionary, yielding a variable-length target sequence for training. In addition, the *aligned phonemes* field encodes categorical outcome annotations for each target phoneme (e.g., /*s*/ → /*t*/, /*iy*/ → /*uw*/), which are labeled as one of four error types: OK (correct), SUB (substitution), INS (insertion), or DEL (deletion).

These error tags were parsed into a parallel supervision sequence aligned at the phoneme level, enabling joint learning of phoneme identity and error events. The resulting paired phoneme and error tag sequences provide dual supervision for the CTC-based multi-task model, supporting simultaneous phoneme transcription and token-level error characterization.

##### 2.5.2.2 Acoustic input representation

Raw audio was resampled to 16 kHz and represented as sequences of 80-dimensional MFCCs, yielding inputs of shape (*T* × 80), where *T* denotes the number of frames (e.g., approximately 108 frames for a typical utterance in the 3–4 year age band). Within each age group, recordings were partitioned at the recording level into training (80%), validation (10%), and test (10%) sets using stratified sampling on speech-status labels to preserve SSD-NP/SSD-P balance.

Prior analyses indicated that clinically meaningful variation in pediatric speech is expressed primarily through temporal dynamics, including the smoothness, speed, and abruptness of acoustic transitions. Accordingly, MFCC sequences were used directly as inputs to a Connectionist Temporal Classification (CTC) framework, which is well-suited for unaligned child speech. CTC was selected because:

1. Children's productions are not strictly aligned to canonical phoneme boundaries. They often include elongated vowels, abrupt stops, or co-articulatory smearing that preclude reliable frame-level phoneme alignment, and CTC does not require frame-level labels.
2. CTC is well-equipped to handle variable-length inputs.
3. CTC's monotonic alignment constraint allows phonetic distortions to span multiple frames without requiring explicit boundary annotations. This design avoids imposing adult-like phonetic regularity assumptions on pediatric speech.

##### 2.5.2.3 CTC-based multi-task model architecture

The core transcription model consists of a two-layer bidirectional LSTM (BiLSTM) acoustic encoder (hidden size 256 per direction) that maps MFCC sequences to contextualized frame-level representations, followed by a linear CTC projection to the phoneme vocabulary augmented with a blank symbol. Bidirectionality enables the encoder to incorporate both preceding and following acoustic context, which is critical for modeling co-articulatory effects and diffuse phoneme boundaries characteristic of child speech.

To support clinically relevant supervision beyond phoneme transcription, we extend the CTC-based encoder with auxiliary task-specific heads trained jointly from shared sequence representations. In addition to the acoustic BiLSTM encoder, phoneme targets are embedded into a 512-dimensional space and processed by a deeper four-layer BiLSTM (hidden size 256), yielding contextual representations of shape (*T* × 512) that serve as inputs to the auxiliary heads.

Specifically, the multi-task extension includes: (1) an error-event classification head that predicts categorical outcome labels (OK/SUB/INS/DEL) at the token level, enabling the model to learn articulatory patterns associated with canonical productions (target) and clinically annotated events (error patterns); (2) an utterance-level disorder classification head that applies mean pooling over sequence representations to predict SSD-NP vs. SSD-P status using weighted cross-entropy to mitigate class imbalance; and (3) a phoneme language-modeling head that performs next-phoneme prediction via cross-entropy on shifted targets, encouraging internal representations to reflect developmentally appropriate phoneme sequencing constraints.

All auxiliary objectives are optimized jointly with the CTC loss, allowing phoneme transcription, error characterization, and disorder sensitivity to be learned from shared temporal representations. This multi-task design encourages the model to learn both fine-grained temporal articulation patterns and higher-level developmental structure intrinsic to pediatric speech.

##### 2.5.2.4 Age-specific training and optimization

Models were trained separately for each age group to reduce confounding from developmental differences in phoneme inventories, articulation timing, and acoustic variability.

The core transcription pathway uses Connectionist Temporal Classification (CTC), which enforces monotonic alignment between acoustic frames and phoneme sequences without requiring frame-level labels. To support auxiliary token-level supervision, phoneme targets were augmented with begin-of-sequence (BOS) and end-of-sequence (EOS) markers, converting a sequence such as [/*d*/, /*ae*/, /*t*/] into [/*BOS*/, /*d*/, /*ae*/, /*t*/, /*EOS*/]. This representation enables autoregressive-style supervision for auxiliary phoneme prediction and error-tagging heads while retaining CTC as the primary acoustic alignment backbone.

All models were trained on 80-dimensional MFCC sequences extracted at a fixed 16 kHz sampling rate. The acoustic encoder consists of a two-layer bidirectional LSTM (hidden size 256 per direction), followed by a linear projection to the phoneme vocabulary plus the CTC blank symbol. Auxiliary heads operate on shared sequence representations: a phoneme-level prediction head, an error-tag head (OK/SUB/INS/DEL), and an utterance-level disorder classifier implemented via mean-pooling over encoder states.

Training was performed for 100 epochs using the Adam optimizer with a learning rate of 1 × 10^−4^. Mini-batches were constructed using padded MFCC sequences, with true frame lengths tracked explicitly to ensure correct CTC computation. Gradients were clipped at a global norm of 5.0 to stabilize optimization on high-variance pediatric speech.

The overall training objective was the unweighted sum of the CTC loss and auxiliary cross-entropy losses:


$$\begin{array}{l} L=L_{\text{ctc}}+L_{\text{lm}}+L_{\text{err}}+L_{\text{cls}}. \end{array}$$

Here, *L*_ctc_ denotes the standard CTC loss over frame-level phoneme distributions, *L*_lm_ is the cross-entropy loss for next-phoneme prediction on BOS/EOS-shifted targets, *L*_err_ is the cross-entropy loss for token-level error tagging (OK/SUB/INS/DEL), and *L*_cls_ is the cross-entropy loss for utterance-level SSD classification. No additional loss re-weighting was applied, allowing the model to jointly learn phoneme alignment, phoneme sequencing, error characterization, and disorder sensitivity from shared representations.

### 2.6 Evaluation

#### 2.6.1 Inference and phoneme sequence decoding

At inference time, the model produces (1) frame-level phoneme logits from the CTC alignment head and (2) token-level phoneme logits from the BOS/EOS-wrapped phoneme decoder. For transcription, CTC logits are greedily decoded by selecting the most likely label at each frame, collapsing consecutive repeats, and removing the blank and padding symbols to yield a variable-length phoneme sequence. For auxiliary analyses (e.g., error tagging and sequencing diagnostics), the decoder's token-level predictions are obtained via greedy argmax over next-phoneme logits, followed by removal of /*BOS*/, /*EOS*/, and /*PAD*/ markers. Predicted phoneme IDs are then mapped back to symbolic phonemes for downstream error analysis.

#### 2.6.2 Evaluation objectives and metrics

Model behavior is evaluated along three complementary dimensions reflecting diagnostic, segmental, and articulatory performance:

1. **Utterance-level disorder classification**. This measures whether a model can distinguish SSD-NP from SSD-P at the utterance level using the sequence-level classifier head. Performance is reported using accuracy, macro-F1, and confusion matrices. Accuracy reflects overall correctness, while macro-F1 balances performance across both classes and is robust to imbalance. In this setting, values near 0.5 indicate chance-level discrimination, whereas values above approximately 0.7 reflect strong model-resolvable diagnostic discriminability.
2. **Phoneme transcription quality**. Transcription fidelity is quantified using Phoneme Error Rate (PER), computed as the normalized edit distance between predicted and reference phoneme sequences:
$$\text{PER}=\frac{\text{EditDistance}\left(\text{hyp},\text{ref}\right)}{\left|\text{ref}\right|}$$
  PER captures substitutions, insertions, and deletions at the sequence level and is reported by age band. A value of 0 denotes perfect transcription; values around 0.3–0.4 indicate moderate error; values above 0.7 reflect highly unstable phoneme recovery. This metric directly quantifies how faithfully the acoustic model reconstructs articulatory structure.
3. **Token-level error event prediction**. Prediction of OK/SUB/INS/DEL events from the error-tag head is evaluated using token-level accuracy (and macro-F1, where reported) computed over non-padding positions, reflecting the model's ability to localize clinically meaningful error patterns.

Together, these objectives capture whether a model can (1) identify disordered speech, (2) reconstruct phoneme sequences, and (3) characterize articulatory deviations.

##### 2.6.2.1 Interpretation of classification and transcription metrics

Throughout this work, classification and transcription metrics are interpreted as properties of model–representation interactions rather than direct measures of child-level speech ability. *Discriminability* denotes the extent to which differences between classes are detectable in a given representation, while *model-resolvable discriminability* refers to the portion of this structure that can be exploited by a specific model under its architectural and training constraints. Accordingly, classification metrics (Accuracy and macro-F1) are treated as measures of model-resolvable discriminability rather than indicators of phoneme category overlap or intrinsic speech competence. Higher values indicate that a model can more consistently exploit structure present in the speech signal under the assumptions of the given pipeline.

In Pipeline A, disorder classification is performed directly from continuous latent acoustic trajectories without explicit phonemic supervision. Performance, therefore, reflects disorder-related information expressed in temporal acoustic dynamics, independent of phoneme identity. In Pipeline B, disorder classification is mediated by phoneme-aligned representations learned via CTC-based sequence modeling, such that classification performance reflects how reliably phonemic structure and error patterns can be extracted from the acoustic signal. Phoneme transcription performance is summarized using phoneme error rate (PER), where lower PER indicates more faithful recovery of the reference phoneme sequence. PER captures within-utterance segmental predictability and should not be interpreted as a measure of diagnostic discriminability.

##### 2.6.2.2 Threshold calibration

For utterance-level disorder classification, predicted probabilities were converted to binary decisions using validation-based threshold calibration rather than a fixed cutoff (e.g., 0.5). For each age-specific model, decision thresholds were selected to maximize Macro-F1 on validation data, accounting for class imbalance and age-dependent prevalence. Threshold calibration was applied strictly at evaluation time and did not influence model training or parameter updates.

##### 2.6.2.3 Developmental interpretation

Before presenting quantitative results, we note an important developmental consideration that informs interpretation across all analyses. Transcription accuracy and disorder classification probe distinct facets of pediatric speech development and are not expected to co-vary monotonically with age. Younger children exhibit broad articulatory variability even in typical speech, inflating sequence-level uncertainty and PER across groups, while SSD-NP and SSD-P productions remain more structurally separable. As children mature, phoneme sequences stabilize, and transcription fidelity improves, but surface realizations of SSD-NP and SSD-P increasingly converge, narrowing diagnostic margins.

#### 2.6.3 Stress-test generalization to ECSC

The ECSC *Frog Story* corpus provides a fundamentally different testbed from CAAP, consisting of long-form, spontaneous narrative speech with no phoneme-level supervision and no time-aligned segmental annotations. Consequently, evaluation on ECSC is necessarily weakly constrained: outputs are examined for decoding stability, qualitative phoneme-sequence structure, and shifts in alignment and error-pattern behavior rather than supervised transcription accuracy. Models trained on CAAP are evaluated on ECSC narratives as an explicit robustness probe under long-form, less controlled speech. The same decoding and metric procedures are applied to quantify how alignment, transcription, and error-pattern predictions shift under naturalistic conditions.

## 3 Results

### 3.1 Data descriptives and dataset contrast (CAAP vs. ECSC)

This section summarizes descriptive properties of the CAAP and ECSC datasets to orient subsequent analyses. We report age-band composition and speech-status balance, followed by contrasts in utterance duration and sequence length that define the distinct evaluation regimes supported by each dataset.

#### 3.1.1 Age-band composition and SSD status balance

**Figure 3:**
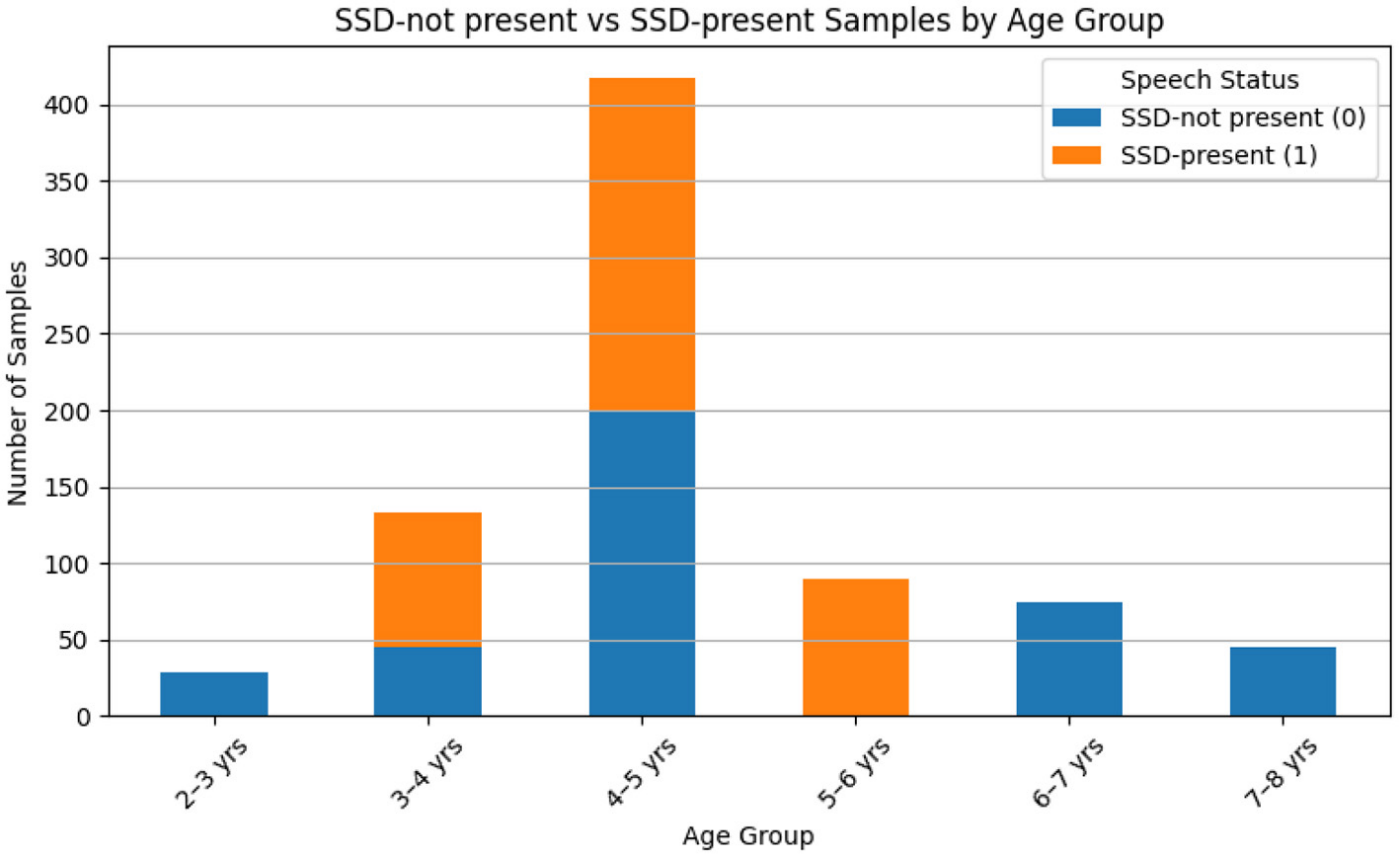
Age-group distribution of SSD-not-present vs. SSD-present samples in the CAAP dataset.

Speech samples were grouped into discrete age bands to support developmentally grounded analysis. Figure 3 shows the distribution of SSD-not-present (SSD-NP) and SSD-present (SSD-P) samples across age groups in the CAAP dataset. In the 3–4 year band, the CAAP dataset comprised 14 samples, and the 4–5 year band had 42 for testing due to the 80% (training), 10% (validation), and 10% (test) case split. The distribution is strongly centered in the 4–5 year band, which accounts for more than half of all samples, while the youngest and oldest groups are comparatively underrepresented, and uneven representation of SSD-P vs. SSD-NP across bands. This non-uniform sampling density reflects typical clinical data collection patterns and reinforces the importance of age-stratified analysis throughout subsequent modeling and statistical comparisons.

#### 3.1.2 Sequence length and duration contrast: CAAP vs. ECSC

The CAAP-derived and ECSC datasets differ substantially in temporal structure and sequence length, reflecting their distinct elicitation paradigms. CAAP recordings consist of short, clinically elicited productions, typically single words or brief utterances, resulting in narrowly distributed durations and compact phoneme sequences. In contrast, ECSC recordings comprise spontaneous, multi-minute narrative speech, yielding substantially longer and more variable sentence durations and phoneme sequence lengths.

**Figure 4:**
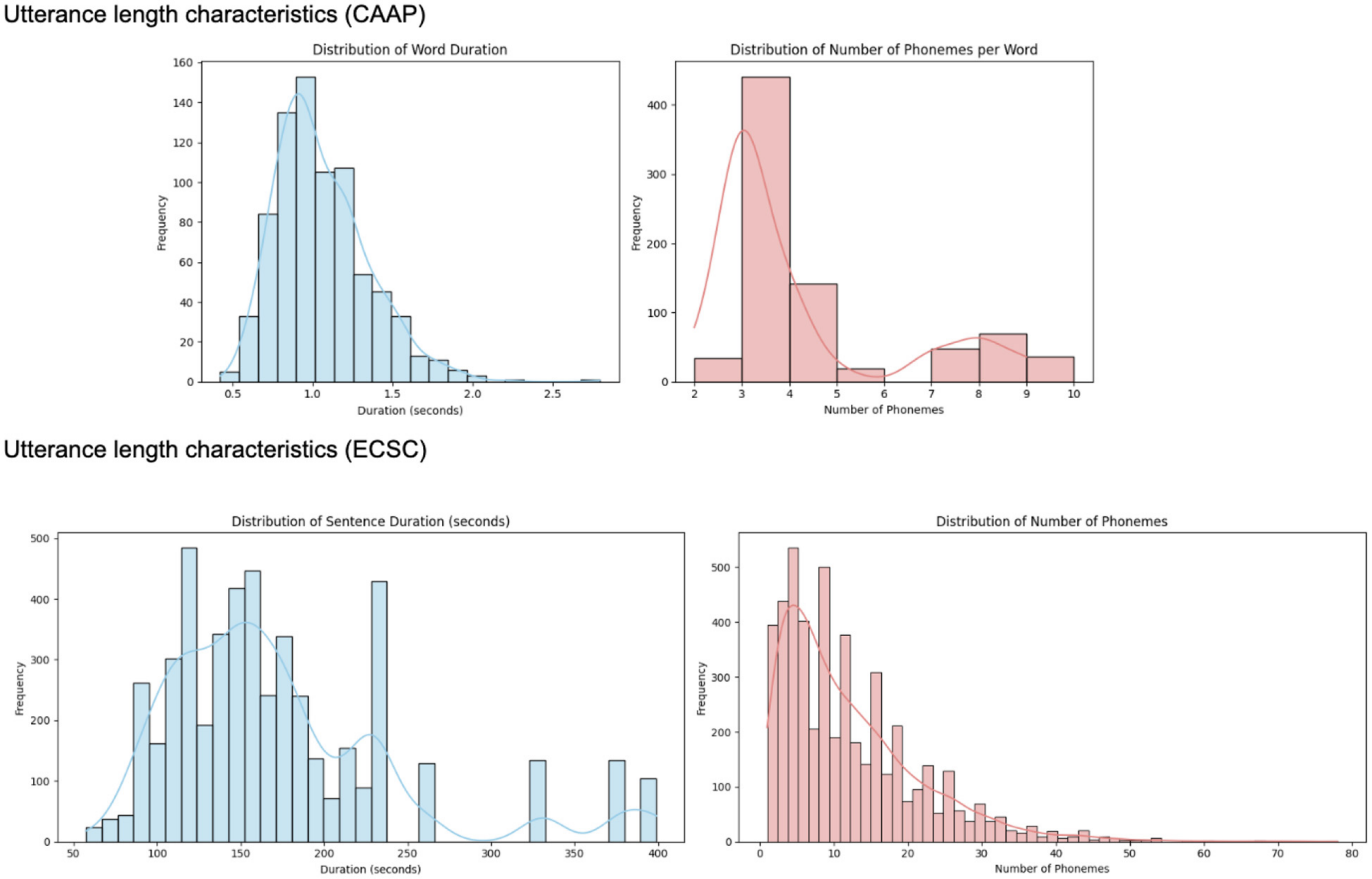
Utterance/sentence-length distribution.

Figure 4 illustrates these structural differences. CAAP utterance durations are tightly concentrated around short time scales, with a corresponding narrow distribution of phoneme counts. ECSC narratives exhibit wide-ranging durations and strongly right-skewed phoneme-count distributions, reflecting naturalistic phrasing, variable speech rate, pauses, and extended prosodic structure.

##### 3.1.2.1 Evaluation constraints for ECSC

These structural differences impose distinct evaluation regimes across datasets. CAAP supports supervised phoneme-level analysis with explicit reference targets, whereas ECSC lacks phoneme-level ground truth and time-aligned segmental annotations. Accordingly, ECSC is used exclusively for robustness and decoding stability analyses rather than supervised transcription accuracy evaluation.

### 3.2 Developmental baseline from clinical annotations

To ground subsequent modeling results in developmentally appropriate reference behavior, we first established phoneme-level baselines derived directly from clinical annotations. These baselines quantified how phoneme production errors were distributed across age and speech status prior to any learned representation or decoding. We evaluated these outputs as candidate biomarker primitives by reporting age-stratified phoneme error structure, coarticulation/transition behavior, and trajectory-based temporal organization alongside model performance metrics.

#### 3.2.1 Age-stratified target–response profiling

**Figure 5:**
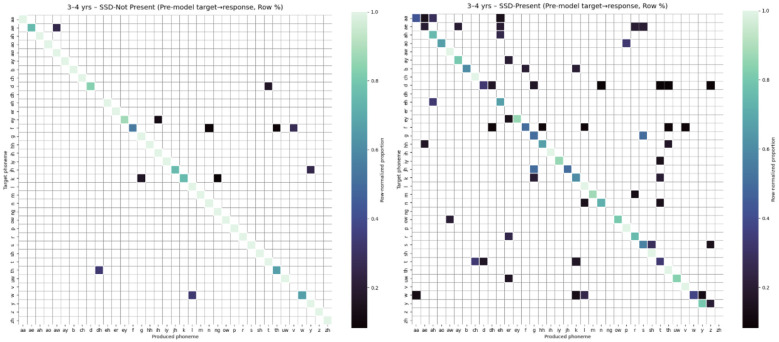
Phoneme confusion matrices for SSD-not present vs. SSD-present for CAAP (3–4 year olds).

**Figure 6:**
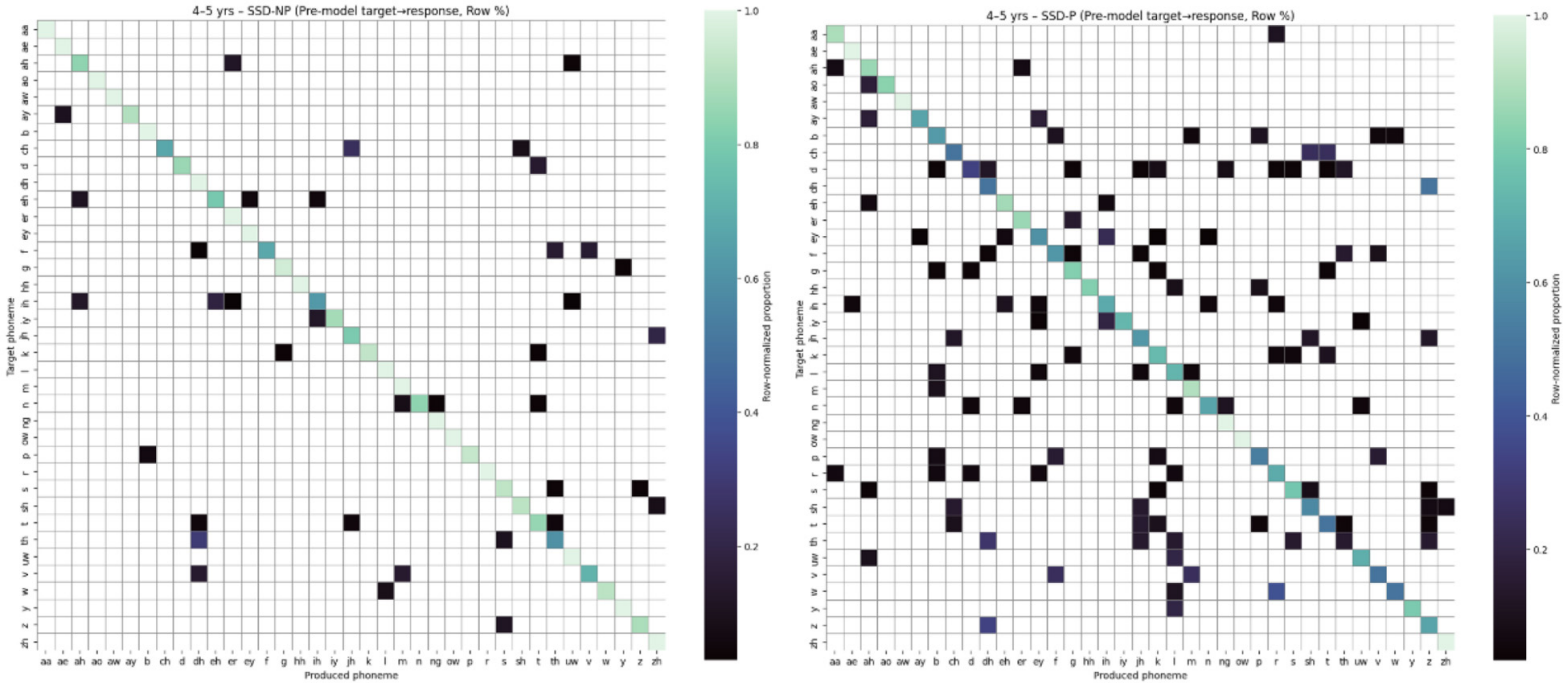
Phoneme confusion matrices for SSD-not present vs. SSD-present for CAAP (4–5 year olds).

Age-stratified target–response profiling provided a developmental baseline for interpreting phoneme confusions. For each age band and elicited target phoneme, we computed row-normalized target → response distributions summarizing (1) the proportion of productions coded as correct and (2) the empirical distribution of categorical outcomes, including substitution, insertion, and deletion events, along with the most frequent substitution responses. Figure 5 illustrates these patterns in the 3–4 year band, and Figure 6 illustrates contrasts in the 4–5 year band. To characterize baseline articulatory error structure independent of any learned model, we first analyzed target–response phoneme mappings derived directly from the CAAP alignment annotations. These matrices reflect empirical substitution patterns observed in children's productions relative to clinically specified targets, prior to any supervised learning or decoding. Rows correspond to intended (target) phonemes and columns to realized responses, capturing the raw phoneme deviation structure present in the dataset itself. In the SSD-NP cohort, counts are concentrated along the main diagonal, consistent with stable target realization mappings across many phonemes. In the SSD-P cohort, diagonal concentration is reduced, and off-diagonal counts are more dispersed, indicating broader and more heterogeneous substitution structure at the population level rather than a single, uniform confusion pattern. These empirical baselines provide a reference against which we later assess whether model-predicted confusion patterns preserve age-appropriate phoneme structure.

### 3.3 Acoustic-prosodic and coarticulation foundations (RQ2)

Acoustic-prosodic and coarticulatory properties of child speech production were characterized before any sequence modeling. These analyses established descriptive, age-aligned foundations for RQ2 by identifying group-level differences and structural regularities in timing, pitch, voicing, and feature interactions that informed subsequent transition-based and temporal modeling.

#### 3.3.1 Group-level summary differences

These comparisons are interpreted descriptively rather than as confirmatory hypothesis tests. Statistical comparisons between SSD-P and SSD-NP groups revealed significant differences in several acoustic-prosodic measures, although the effect sizes were small. Duration differed significantly between groups (*t* = 2.46, *p* = 0.014, Cohen's *d* = 0.175). Pitch-derived rhythmic modulation metrics showed a highly significant group difference (*t* = 4.71, *p* < 0.001, Cohen's *d* = 0.335), and energy-derived rhythmic variability measures also differed significantly (*t* = −2.77, *p* = 0.0058, Cohen's *d* = −0.197). Voicing proportion did not differ significantly (*t* = −1.342, *p* = 0.1801, Cohen's *d* = −0.095). One-way ANOVA across age bands revealed no significant effects for number of peaks, speech rate, or mean inter-peak interval (all *p* ≥ 0.13; lowest *p* = 0.1277), providing no evidence of age-group differences in these rhythmic timing measures within the CAAP task. Because CAAP words are single-word, predominantly monosyllabic, short duration utterances, these features should be interpreted as descriptive acoustic tendencies rather than stand-alone clinically actionable markers.

#### 3.3.2 Feature correlation structure

**Figure 7:**
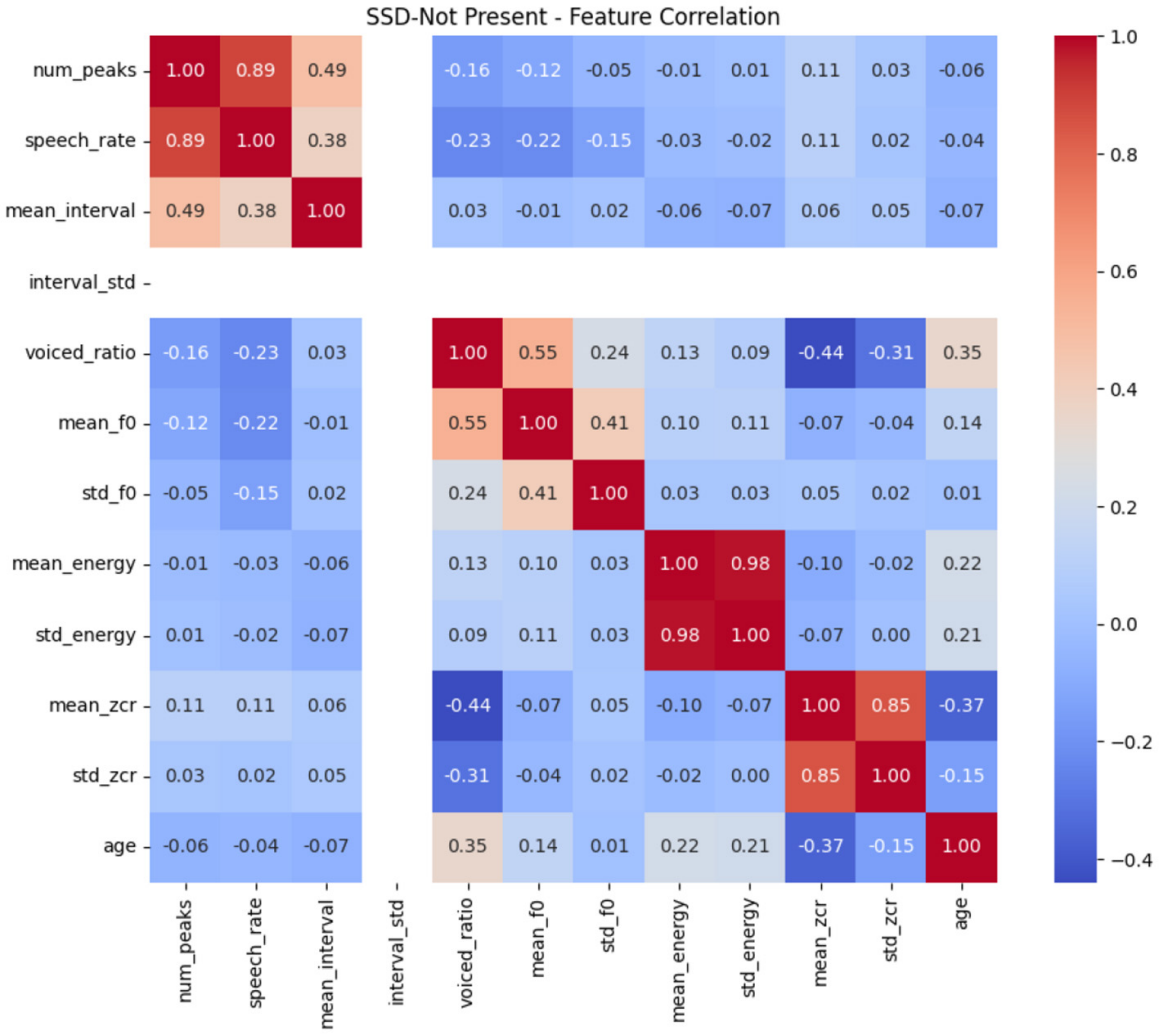
Correlation heatmap of acoustic-prosodic features in the SSD-NP.

**Figure 8:**
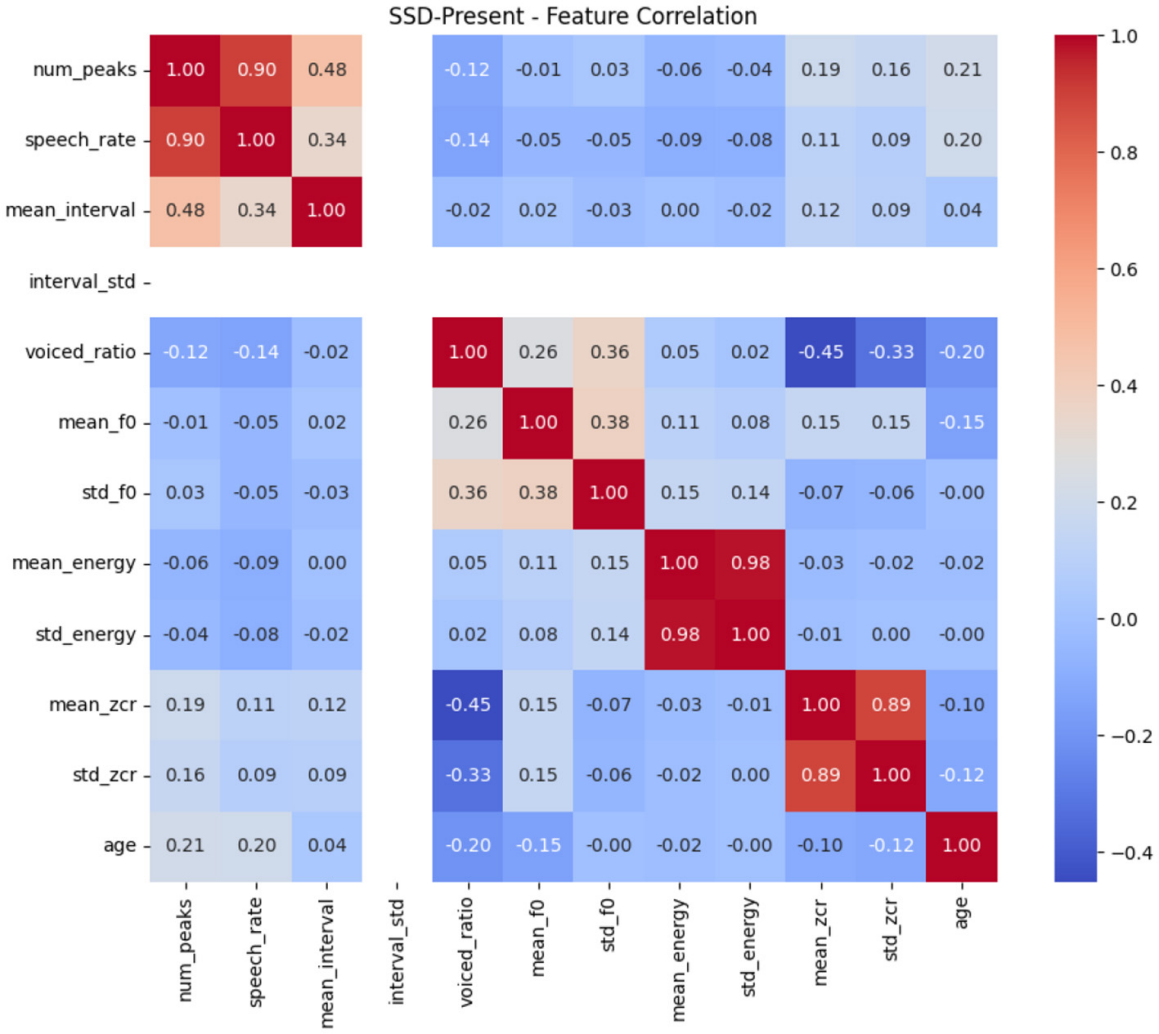
Correlation heatmap of acoustic-prosodic features in SSD-P.

We used within-group feature–feature correlation structure as a descriptive check on whether the acoustic measures behaved as coordinated sets (e.g., voicing with F0; energy with ZCR) vs. behaving more independently. This matters for RQ2 because the coarticulation/transition features are derived from spectral dynamics, and the heatmaps indicate whether other timing/voicing/energy measures co-vary in ways that suggest redundancy or complementary information. Accordingly, we report the correlations to (1) document internal consistency of the extracted measures and (2) motivate treating feature interactions as potentially informative when interpreting transition dynamics. The correlation heatmaps summarized how acoustic features covaried within each speech status group (Figures 7, 8). In the SSD-NP group (Figure 7), voicing and pitch measures were more consistently coupled (voiced ratio ↔ mean F0 = 0.55; mean F0 ↔ F0 SD = 0.410). Age also showed small-to-moderate associations with several features (age ↔voiced ratio = 0.35; age ↔ mean energy = 0.22) and a moderate negative association with mean ZCR (age ↔ mean ZCR = –0.37). In contrast, the SSD-P group (Figure 8) showed weaker coupling among pitch/voicing measures (voiced ratio ↔ mean F0 = 0.26; mean F0 ↔ F0 SD = 0.38). Weak associations were observed between age and pitch/voicing variables (age↔ voiced ratio = –0.20; age ↔ mean F0 = –0.15; age ↔ F0 SD = 0.00). Age showed a weak negative association with mean ZCR in SSD-P (age ↔ mean ZCR = –0.10). These groupwise differences in covariance structure motivated treating feature interactions as potentially developmentally informative in downstream modeling.

### 3.4 Modeling results on clinically elicited speech (RQ1)

#### 3.4.1 Summary of modeling outcomes

Table 1 provides a consolidated overview of age-stratified model performance on the CAAP dataset for disorder classification and phoneme transcription across two modeling pipelines. Interpretation of classification and transcription metrics, including distinctions between Pipeline A and Pipeline B, is described in Section 2.6.2.1.

**Table 1:** Summary of CAAP results across modeling stages, pipelines, and age groups ^*a*^.

Stage	Pipeline	Model/method	Task	Age group	Metric	Result
Temporal modeling	Pipeline A	BiLSTM (latent)	Disorder classification	3–4 yrs	Accuracy 0–1 (1 = perfect)	0.778
				4–5 yrs		0.583
		BiLSTM + Attention		3–4 yrs		0.852
				4–5 yrs		0.631
Phoneme modeling	Pipeline B	CTC + Multi-task	Disorder classification	3–4 yrs	Macro-F1 (opt) 0–1 (1 = perfect)	0.785
				4–5 yrs		0.547
		CTC	Phoneme transcription	3–4 yrs	PER 0–∞ (0 = perfect; lower is better)	0.762
				4–5 yrs		0.381

^*a*^ Pipeline A classifies disorder from latent acoustic trajectories without explicit phoneme modeling, whereas Pipeline B classifies disorder from phoneme-aligned representations learned via CTC-based sequence modeling.

Because the effective test-set sizes within each age-stratified split were small, the reported accuracy and macro-F1 values should be interpreted as preliminary descriptive estimates rather than stable estimates of generalization performance. These values are therefore used to characterize model-resolvable patterns within the current sample, not to establish clinically deployable performance benchmarks.

#### 3.4.2 Pipeline A: latent trajectory disorder classification (BiLSTM ± attention)

We evaluated the latent trajectory modeling pipeline on the CAAP dataset using utterance-level disorder classification. VAE-derived latent acoustic trajectories were modeled with the BiLSTM, with and without an attention mechanism, and performance was assessed separately within each age band.

In the 3–4 year group, the baseline BiLSTM achieved an accuracy of **0.778**. In the 4–5 year group, accuracy decreased to **0.583**. Incorporating temporal attention improved classification performance in both age bands, yielding an accuracy of **0.852** for ages 3–4 and **0.631** for ages 4–5. These results indicate that attention improved disorder classification from latent acoustic trajectories across developmental stages.

**Figure 9:**
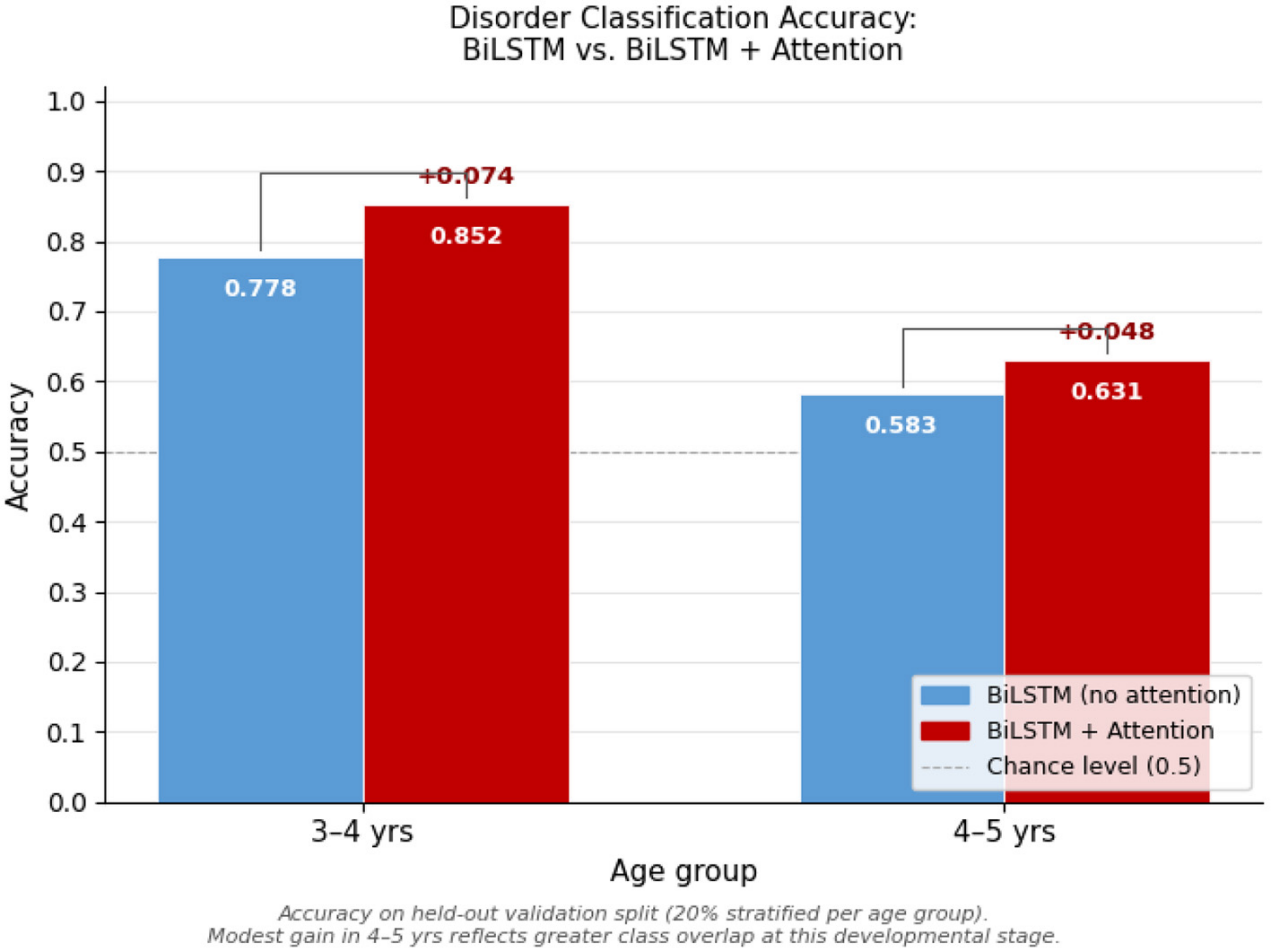
Disorder classification validation accuracy for BiLSTM without and with attention across age groups with sufficient class representation for binary classification (3–4 and 4–5 yrs). Groups 2–3, 5–6, 6–7, and 7–8 yrs contained only one class in the available CAAP sample and are excluded.

Figure 9 compares the validation accuracy of disorder classification for the BiLSTM model with and without attention across age groups with sufficient class representation. Both models were trained with identical stratified splits per age group, with validation monitored for learning rate scheduling. Validation accuracy is reported to enable direct comparison between model variants trained under identical conditions, with results interpreted as relative performance differences rather than absolute generalization metrics. Attention yielded consistent gains in both groups: +0.074 in the 3–4 year group (0.778 → 0.852) and +0.048 in the 4–5 year group (0.583 → 0.631). The larger gain in younger children is consistent with more focal articulatory instability at this stage, where selective weighting of latent trajectory segments provides greater discriminative benefit. The modest gain in 4–5 years reflects greater acoustic overlap between SSD-P and SSD-NP at this age.

#### 3.4.3 Pipeline B: CTC-based phoneme modeling

##### 3.4.3.1 Disorder classification

Utterance-level disorder classification was evaluated using the sequence-level classifier head of the CTC-based multi-task model. Performance is reported using accuracy and Macro-F1, with decision thresholds selected via validation-based optimization. In the 3–4 year group, baseline performance reached an accuracy of 0.643 and a Macro-F1 of 0.626. After threshold calibration, Macro-F1 increased to 0.785, indicating substantial model-resolvable discriminability between SSD-NP and SSD-P speech at this developmental stage. In the 4–5 year group, baseline performance was lower (accuracy = 0.476, Macro-F1 = 0.465). Threshold optimization yielded modest improvement, with Macro-F1 increasing to 0.547, indicating reduced model-resolvable class discriminability relative to the younger age group.

##### 3.4.3.2 Phoneme transcription accuracy

Phoneme transcription quality was quantified using phoneme error rate (PER), computed as the normalized edit distance between predicted and reference phoneme sequences. In the 3–4 year group, the model achieved a mean PER of 0.762, indicating substantial divergence between predicted and reference sequences. In contrast, the 4–5 year group exhibited a markedly lower mean PER of 0.381, consistent with increased segmental stability in older children. PER is reported by age band to support age-aligned comparison.

##### 3.4.3.3 Substitution structure

Phonemes on both axes are ordered alphabetically by their ARPAbet symbol labels; this ordering does not reflect phonetic proximity (e.g., by place or manner of articulation), so visual adjacency in the matrix does not imply phonetic similarity.

To characterize error structure in the phoneme-recognition pipeline, we constructed target → response substitution matrices by comparing decoded phoneme sequences to reference phoneme strings on the held-out-test set. Rows correspond to target phonemes and columns to predicted model responses, with rows normalized by target frequency to match the target → response framing used in the annotation-based baseline analyses. We interpret prominent off-diagonal patterns as evidence of which phonetic distinctions were challenging for the model under pediatric acoustic variability, and we treat correspondence with the clinical baseline as a qualitative consistency check (i.e., whether broad structural trends were similar). These analyses were intended to set the foundation for scalable next-stage validation testing.

**Figure 10:**
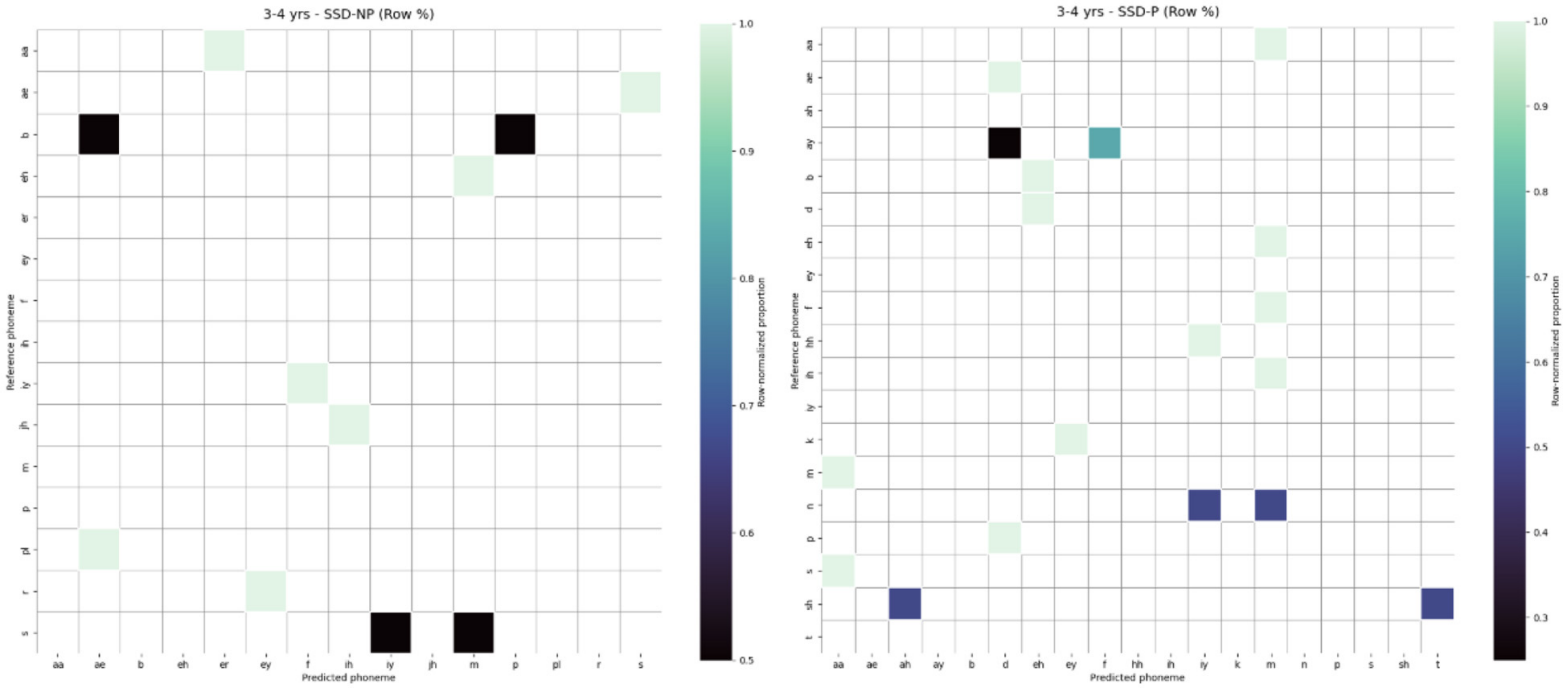
Phoneme substitution matrices for the 3–4 year group, SSD-not present vs. SSD-present, derived from model-decoded phoneme sequences.

As shown in Figure 10, the 3–4 year substitution matrices exhibited relatively diffuse off-diagonal mass, particularly for SSD-P speech. In model terms, indicating reductions in articulatory contrast that span place, manner, and voicing rather than remaining confined to narrow phonetic neighborhoods. In contrast, SSD-NP matrices at the same age remain more diagonally concentrated, consistent with emerging but still variable phonological control.

**Figure 11:**
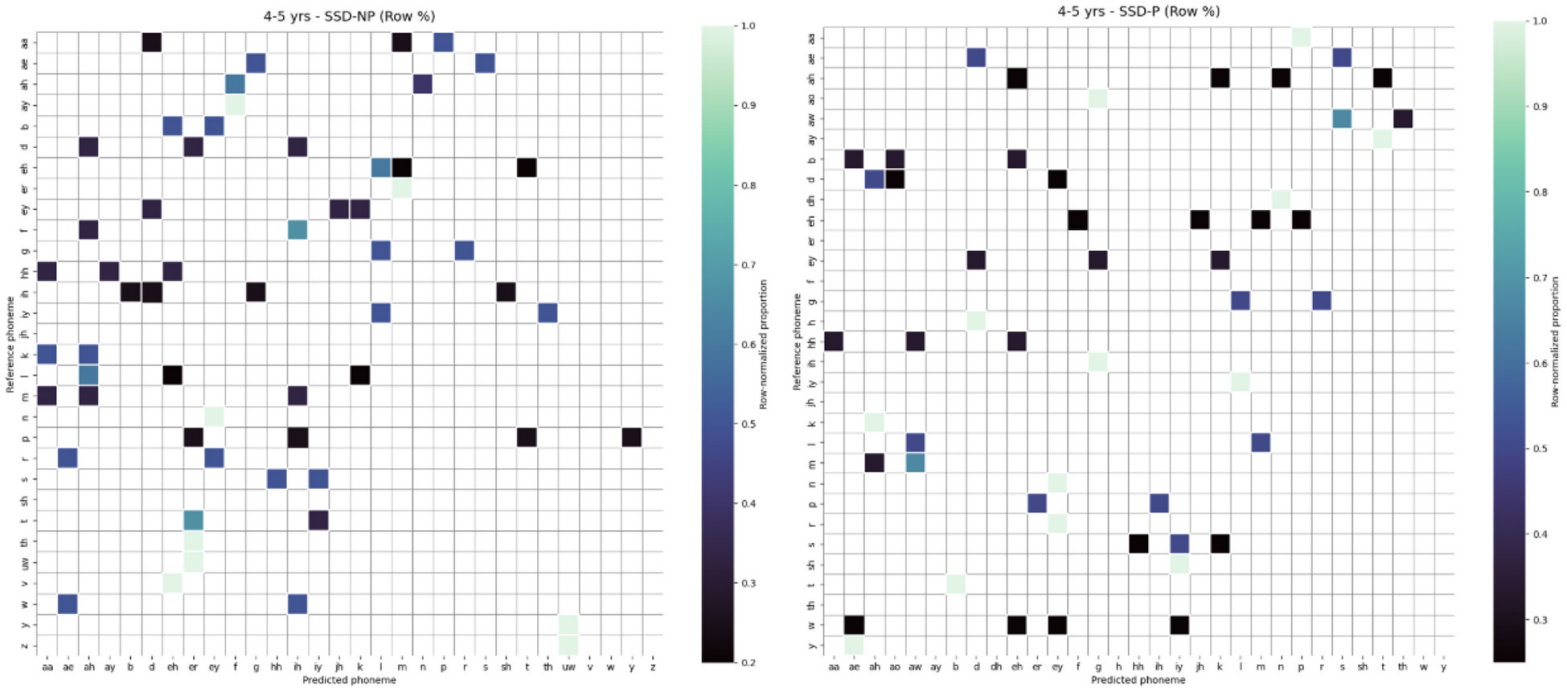
Phoneme substitution matrices for the 4–5 year group, SSD-not present vs. SSD-present, derived from model-decoded phoneme sequences.

In the 4–5 year group, decoded substitutions became sparser and more localized, consistent with the increasing segment stability reflected in lower PER at this age. The corresponding matrices (Figure 11) show sparser off-diagonal entries that cluster around phonetic neighbors, indicating a transition from class-wide collapse to finer-grained contrast confusions. Even in SSD-P speech, deviations at this age are embedded within an otherwise coherent segmental structure, while SSD-NP matrices are strongly diagonal, reflecting stable segmental realization.

Together, Figures 10, 11 demonstrate a sharpening of the error landscape with age. The transition from diffuse, cross-category substitutions to localized, contrast-level confusions mirrors known developmental trajectories in phonological acquisition. Differences between model-predicted and annotation-derived phoneme distributions are expected, as clinical annotations reflect intended targets, whereas model outputs are driven by acoustic discriminability and CTC decoding dynamics, which favor longer and acoustically salient segments. Nonetheless, the organization of the substitution matrices closely parallels age-referenced phonological structure, supporting the interpretability of the model's phoneme-level predictions.

##### 3.4.3.4 Qualitative examples

To supplement quantitative metrics, we examined stratified sample predictions from each age group, focusing on three outputs per utterance: (1) the decoded phoneme sequence, (2) the predicted disorder label, and (3) the associated disorder probability. These examples are presented to illustrate observable model outputs at the utterance level rather than to support statistical inference.

In the 3–4 year cohort, disorder probabilities spanned a wide range for both SSD-not-present and SSD-present samples. For example, an SSD-NP production such as /s iy l/ (“*seal”*) received a disorder probability of 0.725 despite a simple phonotactic structure. Conversely, some SSD-P samples (e.g., /n ay f/) (“*knife”*) were assigned comparatively low disorder probabilities (e.g., 0.284). Decoded phoneme sequences in this age group often exhibited broad substitutions and repeated vowel or nasal segments (e.g., /sh uw/→/uw uw uw/). Across samples, both probability values and decoded outputs showed substantial variability within this cohort.

In the 4–5 year cohort, disorder probabilities were more closely aligned with ground-truth status. SSD-NP productions such as /uw/ (“*zoo”*) yielded moderate, near-classification-threshold probabilities (e.g., 0.329), whereas SSD-P productions containing clear segmental deviations (e.g., /n/) (“*zoo”*) often received high disorder probabilities (e.g., 0.824). In this group, disorder probabilities were less influenced by isolated transcription errors and more consistent across utterances with similar articulatory structure.

Across age groups, decoded phoneme sequences and associated disorder probabilities varied in dispersion. Younger cohorts displayed a broader spread of probability values and more variation in decoded sequences, whereas older cohorts showed narrower probability ranges and more uniform decoding patterns across utterances.

### 3.5 Transition modeling results (RQ2)

#### 3.5.1 Transition magnitude distributions

To quantify co-articulatory strength across development, we analyzed distributions of transition magnitudes derived from MFCC trajectories and their temporal derivatives (Δ, Δ^2^) for CAAP utterances. Transition magnitude summarizes the extent of spectral change across adjacent phonetic regions, with larger values corresponding to sharper, more distinct phoneme boundaries and smaller values indicating smoother or slurred transitions.

Analyses were constrained to the 3–4 and 4–5 year age bands to align with prior stratified modeling results. In both age groups, SSD-not present children exhibited higher mean transition magnitudes than SSD-present children. This separation was strongest in the 3–4 year cohort (independent *t*-test: *t* = 8.553, *p* < 0.001, Cohen's *d* = 1.691) and remained significant, though attenuated, in the 4–5 year cohort (*t* = 2.182, *p* = 0.0297, Cohen's *d* = 0.216). Higher transition magnitudes in SSD-not present samples reflect sharper articulatory contrasts between successive phonemes, whereas lower values in SSD-present samples indicate reduced spectral change across phoneme boundaries.

Group-wise analysis further revealed the following structural properties:

- In both age bands, SSD-P samples exhibited lower mean transition magnitudes (approximately 51–55) compared with SSD-NP counterparts (approximately 57–67), quantifying the group separation described above.
- The magnitude of group separation was substantially larger in the 3–4 year cohort, with a more moderate but still statistically significant difference observed in the 4–5 year cohort.
- Partial overlap between SSD-not present and SSD-present distributions was evident in the 4–5 year group, indicating increasing interaction between developmental variability and disorder severity with age.

**Figure 12:**
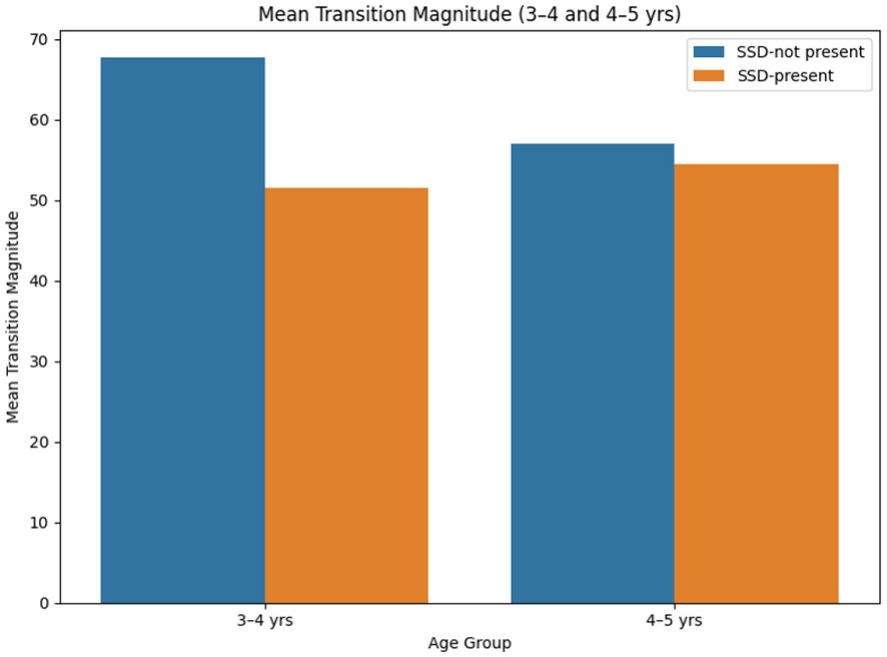
Mean transition magnitudes by age group for SSD-not present and SSD-present children in the CAAP dataset. Higher values indicate sharper spectral transitions between adjacent phonetic regions, reflecting clearer articulatory boundaries.

These distributional trends, summarized in Figure 12, demonstrate that transition magnitude captures a developmentally sensitive dimension of articulatory organization that differentiates typical and disordered speech at the group level, while also highlighting substantial within-age variability.

#### 3.5.2 Sequential modeling of transition dynamics

Because co-articulation unfolds over time, static summary statistics do not capture how transition strength evolves within an utterance. Qualitative inspection of transition-magnitude trajectories revealed that disordered speech was characterized not only by reduced magnitude but also by locally irregular and unstable temporal patterns.

To assess whether the temporal organization of transitions provides additional discriminatory information, transition-magnitude time series were used as inputs to a recurrent sequence classifier trained to predict SSD-NP vs. SSD-P status. The transition-only model achieved approximately 61% training accuracy and approximately 46% validation accuracy. These results indicate that phoneme-joining behavior contains a discriminative signal but is insufficient for robust disorder classification in isolation.

Together, the distributional and sequential analyses demonstrate that co-articulatory dynamics form a meaningful and developmentally sensitive dimension of pediatric speech characterization, while also motivating integration with complementary feature streams (e.g., phoneme substitution structure and prosodic cues).

### 3.6 Out-of-domain robustness on ECSC (RQ3)

#### 3.6.1 Numerical stability on long narratives

When applied to ECSC “Frog Story” recordings, the CTC-based multi-task model produces frame-level phoneme logits over extended narrative sequences and generates recording-level outputs without numerical instability. Despite the substantial mismatch in temporal scale between ECSC narratives and the short, clinically elicited CAAP utterances used for training, model inference remains well-behaved: activations do not diverge, decoding proceeds over multi-minute recordings, and sequence-level outputs are produced for all files. This indicates that the learned acoustic and sequence representations are not tightly coupled to CAAP's short-utterance regime and generalize to long-form, naturalistic child speech at the level of numerical inference.

#### 3.6.2 Decoding failure modes under greedy CTC

Although inference remains numerically stable, raw CTC decoding on ECSC exposes a clear failure mode. Greedy decoding over long narratives yields highly repetitive outputs dominated by the CTC blank symbol, with frame-level predictions collapsing into extended runs of identical labels. Quantitatively, this behavior is reflected in an average blank-frame ratio approaching 1.0 (mean blank ratio ≈0.99999), and an extremely low compression ratio (mean ≈3.6 × 10^−6^). Mean frame-level entropy remained low (mean ≈0.36), indicating confident but degenerate predictions dominated by a single class.

This behavior reflects a known limitation of naive CTC decoding when applied outside the training regime: the model was optimized on short, bounded utterances, and its emission dynamics are not calibrated for multi-minute sequences containing long unvoiced spans, breathing pauses, and narrative-level prosodic variation. As a result, frame-wise maxima frequently converge to the blank class across large portions of the recording, producing degenerate phoneme sequences unless additional constraints or post-processing are applied.

These quantitative decoding metrics confirm the structural mismatch between controlled clinical speech and spontaneous pediatric narratives. While the ECSC stress test demonstrates that the current architecture captures structured phoneme-level representations under controlled elicitation and maintains numerically stable inference under long-form naturalistic speech, it also shows that robust phoneme transcription in realistic child speech requires explicit temporal control mechanisms beyond standard greedy CTC decoding. The implications of this failure mode, along with potential architectural and decoding extensions, are addressed in the Discussion.

Together, the CAAP and ECSC results delineate both the strengths and the current limits of the proposed pediatric phoneme modeling framework. The system successfully learns age-aware phonological structure, preserves interpretable phoneme-level error patterns, and maintains stable inference under long-form naturalistic speech. At the same time, the ECSC robustness analysis reveals that deployable pediatric speech technologies will require additional hierarchical, duration-aware, or constrained decoding strategies to bridge the gap between clinical elicitation and real-world child narratives.

## 4 Discussion

### 4.1 Measurement objectives

This study aimed to establish foundations for pediatric vocal biomarkers by evaluating a developmentally informed phoneme-level recognition and error analysis framework for pediatric speech. Using age-stratified CAAP productions with phoneme-level targets, we implemented two analytical pipelines that shifted emphasis from IPA transcription alone to multi-dimensional, age-conditioned measurement. We operationalized this framework with three coupled components: (1) latent trajectory modeling intended to capture continuous acoustic dynamics that may not be well summarized by categorical phoneme labels, (2) supervised phoneme error profiling (PER, confusion structure, and substitution signatures), (3) coarticulation dynamics quantified as transition behavior in MFCC trajectories and their first and second derivatives, and (4) temporal modeling of learned acoustic representations (VAE latent trajectories with BiLSTMs, with and without attention), alongside supervised phoneme transcription using CTC-based sequence learning.

From a clinical perspective, the measures reported here should be interpreted as quantitative complements to expert speech-language evaluation rather than as diagnostic replacements. Phoneme-level error profiles summarize model- and annotation-derived patterns of phoneme production relative to age-stratified reference patterns; transition magnitude reflects the sharpness or smoothness of spectral-acoustic change between adjacent frames, and latent-space measures provide a compact description of broader speech-pattern organization. Together, these outputs may help identify measurable dimensions of pediatric speech production that warrant closer clinical review. However, they should not be used for screening, diagnosis, or eligibility decisions without further validation in larger and clinically diverse pediatric cohorts. Rather than proposing a diagnostic tool, this study represents an initial step toward evaluating automated, developmentally contextualized metrics for characterizing multiple dimensions of child speech production.

### 4.2 Representation-level evidence for SSD signal

Before interpreting phoneme- and transition-level findings, we first contextualized Pipeline A as a representation-level analysis that addressed a methodological constraint present throughout pediatric modeling: categorical phoneme labels do not fully specify the continuous temporal organization of children's speech productions. In Section 4.4.1 (Table 1) and Section 4.4.2, using latent acoustic trajectories alone produced above-chance discrimination between SSD-P and SSD-NP speech across age bands. These outcomes showed that the CAAP recordings contained model-resolvable disorder-related structure in the global acoustic–temporal organization that was detectable independent of phoneme identity. Pipeline A served as a complementary measurement stage: it provided evidence that disorder-relevant information was present in continuous dynamics that may not be fully captured by transcription-derived variables alone.

### 4.3 Acoustic–prosodic structure

With this representation-level context in place, we next examined which interpretable acoustic dimensions exhibited group-level structure in CAAP productions and were plausibly relevant to developmental timing and coordination. Specifically, SSD-P and SSD-NP differed significantly in utterance duration (*t* = 2.46, *p* = 0.014), pitch-derived modulation metrics (*t* = 4.71, *p* < 0.001), and energy-derived rhythmic variability (*t* = −2.77, *p* = 0.0058), while voicing proportion was not significantly different (*p* = 0.18). These age-stratified results were consistent with observed developmental shifts during the early years of development, showing progressive development of motor coordination and control over time (MacNeilage et al., 2000; Locke, 1983; Vorperian et al., 2005). Relevant deviation may be expressed through temporal coordination and prosodic organization, not solely through categorical segmental outcomes. In our framework, such measures function as *candidate measurement features*: interpretable, quantifiable descriptors of production structure that can be conditioned on age and compared across cohorts.

### 4.4 Divergence between transcription accuracy and SSD discriminability

Across analyses, a consistent developmental pattern emerged: phoneme transcription accuracy and disorder classification do not improve in parallel with age. In CAAP-elicited clinical speech, phoneme transcription improved markedly from ages 3–4 to 4–5 (PER decreasing from approximately 0.76 to 0.38), reflecting increased segmental stability and more predictable acoustic-to-phoneme mappings with maturation. In contrast, disorder classification performance decreased in the 4–5 year band relative to 3–4, indicating greater overlap between SSD-NP and SSD-P speech during mid-childhood.

This divergence reflects a shift in the nature of disorder-related cues across development, consistent with prior accounts of non-linear maturation in speech motor control and phonological organization during early childhood (Kent, 1996; Namasivayam et al., 2020; MacNeilage et al., 2000). In the 3–4 year group, SSD-P speech exhibits broad acoustic–temporal deviations (e.g., diffuse transitions, timing instability, and globally reduced organization), consistent with prior descriptions of early speech development as characterized by high variability and immature coordination across articulatory subsystems (Kent, 1996; Vorperian et al., 2005; Namasivayam et al., 2020). This provided a strong signal for model-based discrimination. By 4–5 years of age, typical development reduces these large-scale differences, and disorder-related cues become more subtle and localized, shrinking the margin between groups in the model's representational space. This pattern is consistent with prior findings showing that children make covert contrasts: distinctions that are instrumentally detectable in the acoustic signal even when adult listeners do not perceive a clear contrast (Namasivayam et al., 2025; Munson et al., 2010). Covert contrast has been described as a stage of acquisition in which productions that sound “merged” perceptually nonetheless contain measurable acoustic separation. Without direct measurement of articulatory gestures, we hypothesize that there are sub-phonemic levels that capture subtle changes as the motor system matures.

Consequently, improved transcription accuracy with age should not be interpreted as increased diagnostic discriminability: transcription metrics primarily capture within-child segmental regularity, whereas disorder classification depends on between-group differences in speech organization, which narrow with development. This refines prior observations in children's ASR (Lee et al., 1999; Potamianos and Narayanan, 2004; Shivakumar and Georgiou, 2020), which document strong age-dependent effects on recognition performance and acoustic variability, by showing that differences relevant to speech disorder likewise vary with developmental stage rather than being uniformly detectable across childhood.

### 4.5 Developmental structure of phoneme error phenotypes

Phoneme-resolved profiling derived directly from clinical annotation provides an interpretable view of how error phenotypes were distributed across age bands. In the 3–4 year band, target → response patterns were less concentrated, with several targets associated with a broader distribution of outcomes. For example, some low vowel and diphthong targets spanned multiple categories(e.g., /aa/ realized as /n/ or /er/). In contrast, target → response structure in the 4–5 year group was more localized with substitution more often concentrated within small phonological neighborhoods (e.g., /l/ realized as /ah/, /eh/, or /ih/). This transition from diffuse, cross-category substitutions to more localized confusions reflects increasing phonological refinement and articulatory stability with development, consistent with accounts of gradient phonological representations and developmental sharpening of contrast structure in children (Munson et al., 2010). Interpreting such patterns relative to age-referenced expectations, rather than adult-like targets, is therefore essential for distinguishing developmentally typical variability from atypical persistence.

### 4.6 Coarticulation dynamics as a complementary measurement dimension

Beyond categorical transcription, coarticulation dynamics captured a complementary acoustic dimension reflecting articulatory coordination. Speech without speech disorder present showed higher transition magnitudes and clearer age-related increases, while speech with speech disorder present exhibited reduced transition contrast at younger ages. These findings are consistent with theories of maturing articulatory precision and suggest that SSD-related differences may manifest not only as phoneme substitutions but also as attenuated acoustic contrast across segment boundaries (Namasivayam et al., 2025).

While transition-based summary statistics (mean, variance, and range) capture global differences in coarticulation strength, they do not represent how articulatory change unfolds within an utterance. In CAAP, inspection of transition trajectories showed that SSD-P speech was characterized not only by reduced magnitude but also by locally irregular patterns. This motivates sequential modeling of transition time series as a complementary lens: it preserves time-resolved structure that may be lost under averaging and provides a bridge between engineered coarticulation descriptors and the temporal sequence models used elsewhere in the pipeline.

### 4.7 Temporal localization of disorder cues and the role of attention

Latent trajectory modeling clarified how disorder-related cues are distributed over time. Adding attention improved disorder classification in both age groups, with a larger gain in the 3–4 year group, where accuracy increased from approximately 0.78 to 0.85. This pattern suggests that diagnostically informative cues are not spread evenly across an utterance, but instead occur in short, salient segments. In younger children, these segments likely correspond to brief episodes of articulatory instability or reduced coordination that are masked when features are averaged over time. By emphasizing such segments, attention provides a practical mechanism for interpretability: it highlights candidate regions of the signal that contribute most to classification decisions and are therefore suitable for targeted clinical inspection.

The attention gains are consistent with a view in which disorder-relevant cues are temporally sparse rather than uniformly distributed. From an interpretability standpoint, this is operationally useful: attention weights nominate short candidate regions for inspection, enabling verification of whether a model decision is driven by localized instability (e.g., abrupt spectral shifts or prolonged low-contrast segments) rather than by diffuse global differences. This aligns with the Introduction's requirement that clinically useful outputs be segment-resolved and verifiable.

### 4.8 Robustness under long-form, naturalistic speech (ECSC)

The ECSC “Frog Story” experiments provide an inference-time stress test of temporal scale and acoustic mismatch. Although ECSC recordings are long narrative segments with no phoneme-level timing and no reference phoneme sequences, the CAAP-trained CTC-based model produces frame-level logits and decoded outputs without numerical instability. This indicates that inference remains well-behaved under extended durations, pauses, and variable prosody. However, because ECSC lacks phoneme-level ground truth in this study, these results do not establish transcription validity; they only characterize decoding stability and qualitative failure modes under out-of-domain conditions.

Taken together, these results demonstrate that phoneme-level modeling in pediatric speech can be both interpretable and technically stable across controlled clinical recordings and naturalistic narrative speech. The successful convergence of sequence models on ECSC narratives, alongside age-resolved error and coarticulation analyses in CAAP, supports the feasibility of building developmentally grounded phoneme-level analytics that extend beyond short, tightly elicited utterances.

### 4.9 Vocal biomarkers as measurement constructs

Given that the preceding analyses instantiate these measures as vocal biomarkers, it is important to clarify how this term is used in the present work. Here, vocal biomarkers are not intended as diagnostic surrogates or disorder labels, but as foundational measurement constructs: quantitatively derived and developmentally informed speech features that make latent production structure observable at scale. The analyses presented in this work operate at the level of measurement rather than inference about etiology or clinical status. Their value lies instead in providing reproducible, interpretable evidence that can be aggregated, compared across age, and integrated with complementary clinical information.

### 4.10 Limitations and measurement scope

At the same time, the present findings should be interpreted within the scope of the available data and measurement choices. Since pediatric dataset are scarce and without standardized recording protocols, interpretability of findings may be influenced by differences in recording conditions. The CAAP-derived samples are unevenly distributed across age, with older groups dominated by typical speech, which may accentuate apparent developmental convergence in the 4–5 year range. CAAP and ECSC also differ substantially in elicitation and annotation, and while stable training on ECSC demonstrates robustness to long, weakly aligned speech, phoneme-level analytics derived from narratives warrant further validation. The ECSC dataset was obtained from an older group of children compared to the CAAP samples, which could have influenced our findings for RQ3. In addition, MFCC-based transition measures provide an interpretable proxy for articulatory coordination but remain indirect and may reflect speaker or recording variability. More broadly, although integrating coarticulation dynamics and latent temporal modeling reduces reliance on categorical transcription alone, surface phonetic patterns cannot uniquely identify underlying planning or motor mechanisms. Within these bounds, the results motivate a clinically aligned measurement strategy that prioritizes complementary evidence over single-score optimization. Age-referenced phoneme error profiles, coarticulation dynamics, and temporally localized instability together provide verifiable signals that mirror how clinicians reason about developmental speech patterns.

A central contribution of this study is the explicit age-aware framing of phoneme-level analytics. Rather than treating variability as noise around an adult reference, the proposed framework embeds developmental expectations directly into measurement, allowing the same phoneme-level deviation to be interpreted differently depending on age. Accordingly, the outputs of this pipeline are best understood as *developmentally contextualized* measurements, not clinical decisions. Future extensions should continue to standardize this narrative by emphasizing age-conditioned reference distributions, continuous age modeling, and uncertainty-aware deviation profiles, while resisting pressure to collapse these signals into single risk scores. Such restraint is essential if phoneme-level analytics are to remain scientifically grounded, extensible to longitudinal data, and suitable as building blocks for downstream clinical or research workflows rather than endpoints themselves.

### 4.11 Code and software availability

All analyses were implemented in Python. Acoustic feature extraction used librosa and torchaudio; statistical analyses and preprocessing used NumPy, pandas, SciPy, and scikit-learn; neural models were implemented using TensorFlow/Keras and PyTorch; and figures were generated using matplotlib and seaborn. The analysis code used for feature extraction, model training, evaluation, and figure generation is available from the corresponding author upon reasonable request. Raw pediatric speech recordings cannot be redistributed by the authors and remain subject to the original dataset permissions and participant privacy restrictions.

## Data Availability

The original contributions presented in the study are included in the article/supplementary material, further inquiries can be directed to the corresponding author/s.
